# Gene Expression Profile of Colon Mucosa after Cytotoxic Insult in wt and* Apc*-Mutated Pirc Rats: Possible Relation to Resistance to Apoptosis during Carcinogenesis

**DOI:** 10.1155/2016/1310342

**Published:** 2016-10-20

**Authors:** Angelo Pietro Femia, Cristina Luceri, Maura Lodovici, Stefania Crucitta, Giovanna Caderni

**Affiliations:** NEUROFARBA Department, Section of Pharmacology and Toxicology, University of Florence, 6 Viale Pieraccini, 50139 Florence, Italy

## Abstract

*Apc*-mutated Pirc rats, spontaneously developing intestinal tumours, are resistant to 1,2-dimethylhydrazine- (DMH-) induced colon apoptosis. To understand this phenomenon, we analyzed the expression of genotoxic stress-related genes* Mgmt*,* Gsta1*, and* Gstp1* in the colon of wt and Pirc rats in basal conditions and 24 h after DMH; plasmatic oxidant/antioxidant status was also evaluated. After DMH,* Mgmt* expression was increased in both genotypes but significantly only in wt rats;* Gsta1* expression was significantly increased in both genotypes.* Gstp1* expression did not vary after DMH but was lower in Pirc rats. Moreover, for each genotype, we studied by microarray technique whole gene expression profile after DMH. By unsupervised cluster analysis, 28 genes were differentially modulated between the two genotypes. Among them were interferon-induced genes* Irf7*,* Oas1a*,* Oasl2*, and* Isg15* and the transcription factor* Taf6l*, overexpressed in DMH-treated wt rats and unchanged in Pirc rats. RT-PCR confirmed their overexpression in DMH-treated wt rats and showed a slighter variation in DMH-treated Pirc rats. Taken together, despite a blunted induction of* Irf7*,* Oas1a*, and* Mgmt*, defective apoptosis in Pirc rats 24 h after DMH is not mirrored by major differences in gene expression compared with wt rats.

## 1. Introduction

Failure to properly respond to cytotoxic insults (resistance to apoptosis) has been linked to carcinogenesis in various organs including colon [1]. During the early phases of the carcinogenesis process, damaged cells which would be eliminated by efficient apoptosis may survive and proliferate into a preneoplastic clone. In the later stages of cancer development, an altered apoptotic response may lead to resistance to cytotoxic chemotherapic drugs.* Apc* gene, mutated in Familial Adenomatous Polyposis (FAP) and in the majority of sporadic colon cancers, has been linked to defective apoptosis [2], but the mechanisms underlying its involvement in apoptosis are not fully understood [3]. Recently [4], we showed that Pirc rats, mutated in* Apc* and spontaneously developing large and small intestinal tumours [5], are resistant to apoptosis induced by 1,2-dimethylhydrazine (DMH), an alkylating chemical also inducing oxidative damage [6, 7], which is widely used to induce colon cancer in rodents [8]. Indeed, 24 h after DMH treatment, Pirc rats showed lower apoptosis when compared to wt rats and failed to stop colon proliferation as wt rats do [4]. To understand the mechanisms through which* Apc* gene affects the response to DMH, we thought it of interest to study in wt and Pirc rats the expression of some genes involved in repair of DNA damage and in the detoxification of chemicals, namely,* Mgmt (O6-methylguanine-DNA methyltransferase)*,* Gsta1 (glutathione-S-transferase-a1)*, and* Gstp1 (glutathione-S-transferase-p1)* in basal conditions and 24 h after DMH. Moreover, to have a comprehensive view of the responses induced by DMH and not only those foreseen in advance, we also studied at whole genomic level the transcription profile after DMH in the two genotypes. In addition, since the ability to respond to DMH may be due, at least in part, to the antioxidant activity present in the plasma, we also measured the antioxidant potential in the plasma of these animals.

## 2. Materials and Methods

### 2.1. Animals and Treatments

Pirc (F344/NTac-Apc^am1137^) and wild type (wt) Fisher F344/NTac rats were originally obtained by Taconic (Taconic Farms, Inc., USA) and bred in CESAL (University of Florence, Italy) in accordance with the Commission for Animal Experimentation of the Italian Ministry of Health. The Pirc colony was maintained by mating heterozygous Pirc rats with wt and pups genotyped at one month of age [4]. Rats were maintained in polyethylene cages under an experimental protocol approved by the Commission for Animal Experimentation of the Italian Ministry of Health.

### 2.2. Experimental Design

Male rats of 2 months of age (both genotypes) were randomly allocated to 1,2-dimethylhydrazine (DMH) treatment (75 mg/kg; s.c.) (DMH-treated rats) or saline (rats in basal conditions) and sacrificed 24 h later as described [4]. The total number of animals used for the experiments was 12, 17, 10, and 14 for wt rats in basal conditions, DMH-treated wt rats, Pirc rats in basal conditions, and DMH-treated Pirc rats, respectively. To minimize the number of animals, we used, when possible, the same animal for different determinations.

At sacrifice, blood was collected for the determination in the plasma of ferric reducing ability of plasma (FRAP) as previously described [4]. The entire colon (from cecum to anus) was dissected and flushed with cold saline. Apparently, morphological normal mucosa (NM) was scraped and stored in RNA-later™ (Qiagen) at −80°C until RNA extraction, carried out by using NucleoSpin® RNA (Macherey-Nagel) according to manufacturer's instructions.

### 2.3. Semiquantitative RT-PCR

Gene expression was evaluated in the colonic NM. Pirc and wt rats were analyzed with procedures previously described [9]. The primers used for the amplification of the different genes were the following:* Gsta1* (also known as Gsta3, NM_031509): Fw (5′-3′) GGACAAAGCAAGGAACCGTT, Rv (5′-3′) CAGAGGGAAGTTGGCCAAAG;* Gstp1* (NM_012577): Fw (5′-3′) TACTTCATCGTCCACGCAGC, Rv (5′-3′) GGACTTGAGCGAGCCTTGAA;* Irf7* (NM_001033691): Fw (5′-3′) CCTCTGCTTTCTGGTGATGC, Rv (5′-3′) GCGCTCAGTCATCAGAACTG;* Mgmt* (NM_012861): Fw (5′-3′) GCCTATTTCCACGAACCTGC, Rv (5′-3′) CCTCATCGCTCCTCCTACTG;* Oas1a* (NM_138913): Fw (5′-3′) CTGAAGAGTCTCATCCGCCT, Rv (5′-3′) CCCTGAGCTGTGTTGAACTC;* Oasl2* (NM_001009682): Fw (5′-3′) GTGAAAAGTCGCCCGGTTAA, Rv (5′-3′) CTGTACCCATCTCCCAAGCA;* Taf6l* (NM_001107575) Fw (5′-3′) AGGACTTCAACAGGGCTCTC, Rv (5′-3′) AGACATGAACTCTGACGGCT;* Isg15* (NM_001106700) Fw (5′-3′) ATCCTCTGAGCATCCTGGTG, Rv (5′-3′) GTGGGGTGTTAGGCCATACT;* Rplp1* (NM_001007604): Fw (5′-3′) TGCTCTCATTAAAGCAGCTGG, Rv (5′-3′) AAAGACCAAAGCCCATGTCA. For each gene, the relative amount of mRNA in the samples was calculated as the ratio of each gene to the ribosomal housekeeping gene* Rplp1* mRNA.

### 2.4. Statistical Analysis

Data for each group (wt rats in basal conditions, DMH-treated wt rats, Pirc rats in basal conditions, and DMH-treated Pirc rats) were expressed as mean percentage (+SE) of the wt rats in basal conditions. To take into account the effect of both genotype and DMH treatment, values were subjected to two-way ANOVA. Analyses were performed with the software GraphPad Prism® version 5.02. Within each genotype, differences between DMH-treated animals and basal conditions were also evaluated by* t*-test, as* post hoc* analysis. Differences were considered significant when *P* was ≤0.05.

### 2.5. Microarray Experiments

For gene expression analysis, total RNA was extracted from samples of colon mucosa harvested from 6 DMH-treated wt rats and 8 wt rats in basal conditions, 4 DMH-treated Pirc rats, and 5 Pirc rats in basal conditions. For each genotype, the labeling and hybridization steps were carried out according to the Agilent protocol (Two-Color Microarray-Based Gene Expression Analysis version 5.7), using a two-color microarray protocol in which DMH-treated samples were contrasted within each genotype with a reference RNA obtained by pooling equal amount of RNA from colon mucosa samples harvested from rats in basal conditions. In the case of wt rats, 6 DMH-treated rats were individually contrasted with a pool from 8 wt rats in basal conditions; in the case of Pirc rats, a pool from 4 DMH-treated rats was contrasted with a pool from 5 Pirc rats in basal conditions performing two technical replicas. The labeled samples were hybridized to Agilent Rat GE 8x60K Oligo 60-mer microarrays, in Agilent microarray chambers (G2534A) at 65°C for 18 h. After hybridization, the microarray slides were washed by using the Agilent Gene Expression Wash Buffers. Fluorescent signal intensities were detected by using the Agilent Scan Control 7.0 software on an Agilent microarray scanner, at a resolution of 2 *μ*m. Image analysis and initial quality control were performed using Agilent Feature Extraction software v9.5. Values for control spots and spots that did not meet the quality criteria were flagged. Quality criteria included a minimal spot size, a median/mean ratio of at least 0.9 for each spot, nonsaturated intensity for both channels, a signal well above background, and a minimal signal intensity for at least one channel. In wt rats, differences in gene expression between DMH treatment and basal conditions were analyzed by* t*-test, comparing normalized red (DMH-induced samples) versus green (basal conditions) signals and applying a cut-off of fold change ≥3. In the case of Pirc rats, since the data came from a pool of samples,* t*-test analysis was not performed and we only applied a cut-off of fold change ≥3. The pairwise average-linkage cluster analysis was applied to differentially expressed genes using the Cluster 3.0 and Treeview software, a method of unsupervised hierarchical clustering in which relationships among genes and samples are represented by trees whose branch lengths reflect the degree of similarity between genes and samples [10]. The microarray data sets supporting the results of this article are available in the MIAME public database ArrayExpress repository [http://www.ebi.ac.uk/arrayexpress/] (accession number: E-MTAB-4910).

## 3. Results

### 3.1. Expression of Genes Involved in DMH Response

**Figure 1:**
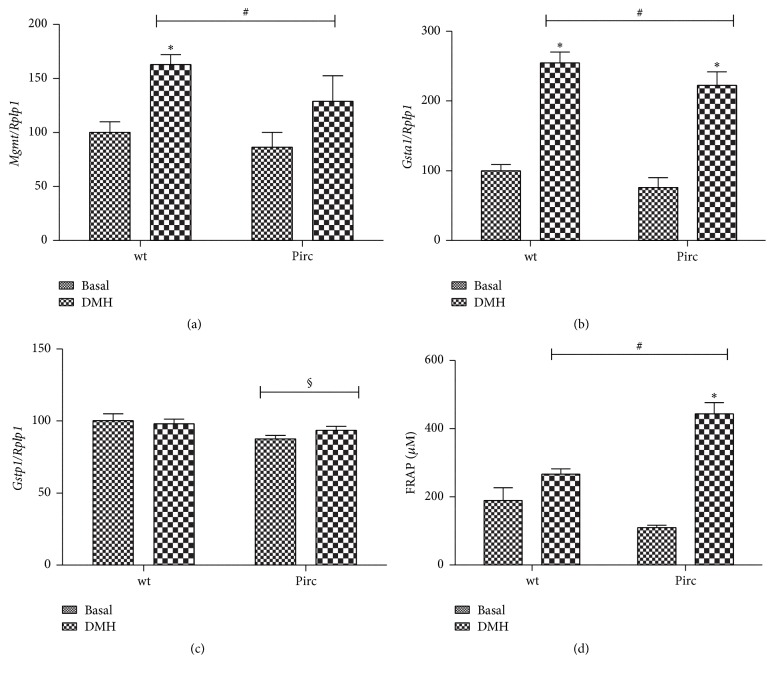
Ratio of* Mgmt*,* Gsta1*, and* Gstp1* ((a), (b), and (c), resp.)/to the ribosomal housekeeping gene* Rplp1* mRNA in wt rats in basal conditions, DMH-treated wt rats, Pirc rats in basal conditions, and DMH-treated Pirc rats. Values are expressed as percent over the basal wt mean values. (d) FRAP determination in wt, DMH-treated wt, Pirc, and DMH-treated Pirc rats. # and §: significant effect of DMH treatment or genotype, respectively, by two-way ANOVA analysis. *∗*: significantly different compared to the corresponding basal condition by* t*-test analysis (*P* < 0.05).

We first studied, by RT-PCR, the expression of* Mgmt*, involved in the response to DMH [11]. Since in the experimental scheme adopted both genotypes were present in basal conditions and after treatment with DMH, data (Figure 1(a)) were analyzed with two-way ANOVA to highlight, if any, an effect of the genotype irrespective of DMH or of DMH treatment irrespective of genotype. The results showed significant upregulation of* Mgmt* expression in the colon mucosa after DMH, while the effect of genotype (wt or Pirc) was not significant (Figure 1(a)). Considering separately each genotype, we noted that* Mgmt* expression was significantly higher in DMH-treated wt rats compared with their basal values (*t*-test), while considering Pirc rats DMH effect was not significant. Regarding* Gsta1* (Figure 1(b)), it was upregulated in DMH-treated rats of both genotypes, while* Gstp1* (Figure 1(c)) expression was not varied by DMH but a significant effect of the genotype was present, with Pirc rats showing a lower expression of this gene compared with wt rats.

We also measured FRAP in the plasma of the rats in the four experimental groups (Figure 1(d)). Two-way ANOVA showed a significant effect of DMH treatment and a significant interaction between strain and treatment. Considering the data for each genotype, DMH-treated Pirc rats showed a higher level of FRAP compared with their basal values, while the DMH effect in wt rats was not significant.

### 3.2. Microarray Experiments

To have a more comprehensive view of the differences between the two genotypes in response to DMH, we evaluated by whole genome array analysis DMH-related changes in the gene expression of colon mucosa comparing six wt rats exposed to DMH (24 h before) with a pool of eight wt rats in basal conditions. The results of this analysis showed that in DMH-treated rats 3173 genes were differentially expressed (*P* < 0.05) compared with rats in basal conditions: 1536 of them were upregulated and 1637 were downregulated. Using an arbitrary cut-off of 3 as fold change (FC) versus baseline, we found 101 genes differentially expressed after DMH (61 upregulated and 40 downregulated) (Supplementary Table  1 in Supplementary Material available online at http://dx.doi.org/10.1155/2016/1310342).

As a second step, we analyzed the expression profile of Pirc rats after treatment with DMH, comparing the expression profile of a pool of colonic samples from 5 rats exposed to DMH (24 h before) with a pool of 4 samples from Pirc rats in basal conditions. In this case, having only two technical replicas, we applied the arbitrary fold change cut-off of 3 as in wt rats, but* t*-test was not performed. This analysis identified 223 genes upregulated and 209 genes downregulated by DMH in Pirc rats (Supplementary Table  2).

**Figure 2:**
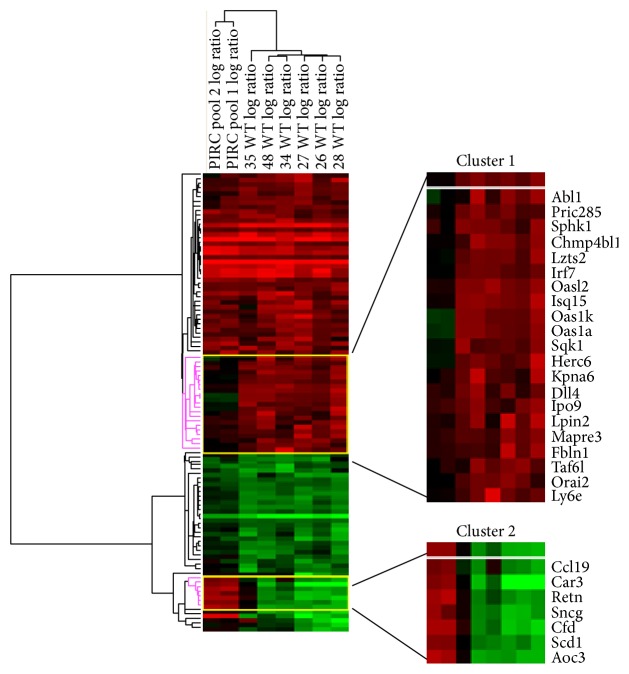
Unsupervised hierarchical cluster analysis based on the 101 genes significantly changed in the wt strain (*P* < 0.05) after DMH and with FC ≥ 3 by microarray technique. Genes are listed in rows. In columns, the individual expressions of 6 DMH-treated wt rats (numeric code) or 2 pooled Pirc RNA are reported. Cluster 1 comprises 21 genes that were overexpressed in wt rats but not changed in Pirc rats; cluster 2 comprises 7 genes that, after DMH treatment, were downregulated in wt rats and overexpressed in Pirc rats. Note that the unsupervised analysis was able to separate Pirc from wt rats (columns).

To highlight differences between the two genotypes, we then performed an unsupervised cluster analysis of the array data relative to the 101 genes significantly changed in wt rats (Supplementary Table  1). With this analysis, relationships among genes and samples are represented by trees whose branch lengths reflect the degree of similarity between the variables (Figure 2). Regarding the similarities among samples, the analysis identified two main clusters composed of Pirc and wt rats, respectively (Figure 2). Regarding the similarities among genes, the analysis identified two main clusters composed of genes predominantly upregulated or predominantly downregulated, respectively. Within the cluster of the predominantly upregulated genes, we noticed a group of closely related genes, cluster 1, composed by 21 genes which were overexpressed in wt rats but not changed in Pirc rats. Within the cluster of the predominantly downregulated genes, we identified a group of genes with similar expression patterns, cluster 2, containing 7 genes which, after DMH treatment, were downregulated in wt rats and overexpressed in Pirc rats (Figure 2).

**Figure 3:**
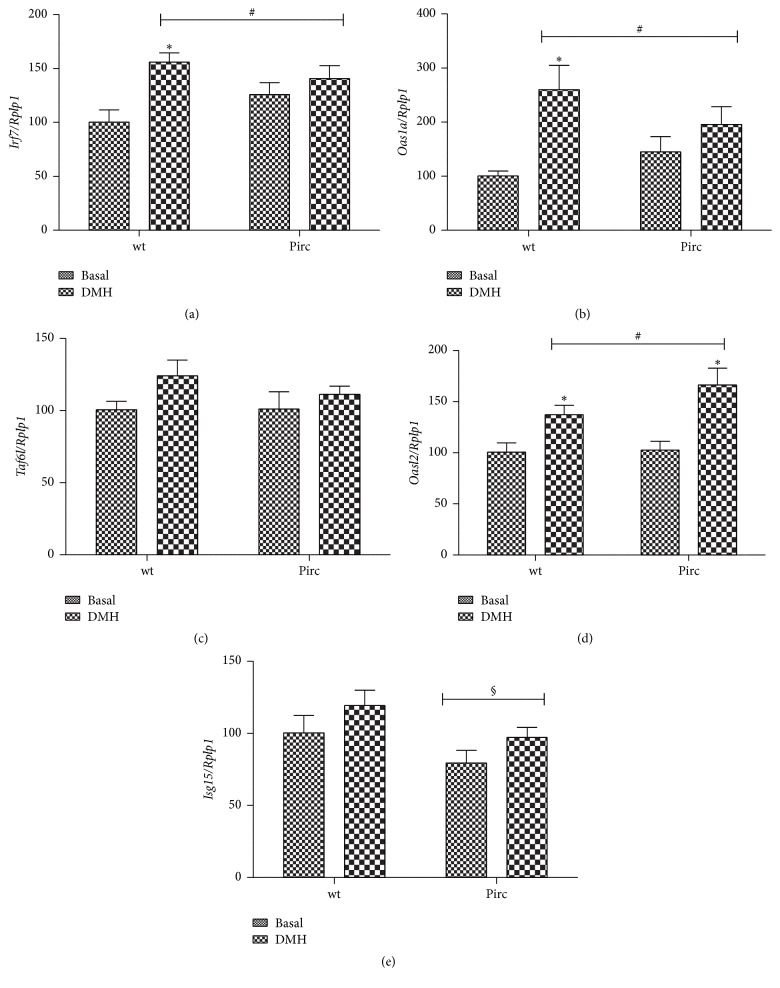
Ratio of selected genes/to the ribosomal housekeeping gene* Rplp1* mRNA in wt rats in basal conditions, DMH-treated wt rats, Pirc rats in basal conditions, and DMH-treated Pirc rats. Values are expressed as percent over the basal wt mean values. (a)* Irf7*; (b)* Oas1a*; (c)* Taf6l*; (d)* Oasl2*; and (e)* Isg15*. # and §: significant effect of the DMH treatment or genotype, respectively, by two-way ANOVA analysis. *∗*: significantly different compared to the corresponding basal condition by* t*-test analysis (*P* < 0.05).

Within cluster 1, we noticed some genes controlled by interferon (IFN) such as* Irf7* (interferon regulatory factor 7),* Oas1a* (2′-5′-oligoadenylate synthetase 1A),* Oasl2* (2′-5′-oligoadenylate synthetase-like 2), and* Isg15* (ISG15 ubiquitin-like modifier) as well as the transcription factor* Taf6l* (TAF6-like RNA polymerase II, p300/CBP-associated factor (PCAF)) which, after DMH, were overexpressed in wt rats but not changed in Pirc rats. For these genes, we verified the results obtained with the arrays with RT-PCR. Regarding* Irf7* gene (Figure 3(a)), RT-PCR experiments confirmed significant upregulation in DMH-treated wt rats when compared with their basal counterpart, while in Pirc rats, the slight upregulation in DMH-treated rats was not significant. Similar upregulation in DMH-treated wt rats was observed for* Oas1a* (Figure 3(b)), with a borderline effect (*P* = 0.08) for* Taf6l gene* (Figure 3(c)), while the variation in DMH-treated Pirc rats was not significant. Regarding* Oasl2* gene (Figure 3(d)), it was upregulated in DMH-treated wt rats but, at variance with the data in the array experiment, was significantly upregulated also in DMH-treated Pirc rats. For* Isg15* gene (Figure 3(e)), only a slight increase in the expression was observed in both wt rats and Pirc rats treated with DMH. The two-way ANOVA of the results shown in Figure 3 showed a statistically significant effect of the DMH treatment (irrespective of the genotype) for* Irf7*,* Oas1a*, and* Oasl2* genes and borderline effect of DMH for* Taf6l* and* Isg15* genes (*P* = 0.06 and 0.07, resp.). For* Isg15* gene, a significantly different expression between the two genotypes was also observed.

## 4. Discussion

DMH (1,2-dimethylhydrazine) is a procarcinogen which is metabolized into methylazoxymethanol (MAM) and in turn to a highly reactive electrophile (methyl diazonium ion), which not only alkylates DNA but also causes oxidative stress [6, 7, 12–14]. This chemical is widely used not only to induce colon carcinogenesis in rodents [8] but also to study the early response to carcinogens in the colon [15, 16]. Using the latter experimental scheme, we recently showed that Pirc rats, mutated in* Apc* gene, were resistant to DMH-induced apoptosis when compared to wt rats [4].

Aiming at identifying genes whose differential expression in wt and* Apc*-mutated rats might explain the defective apoptosis in Pirc rats; we studied* Mgmt*, involved in the response to alkylating agents, as well as* Gsta1* and* Gstp1*, involved in the defence against xenobiotic/carcinogens [11, 17]. MGMT is a key enzyme in the repair of DNA damage by alkylating agents, acting with a suicidal mechanism [11]. Although Kerr and colleagues [16] showed downregulation of* Mgmt* expression in rat colon 6 h after azoxymethane (AOM), a metabolite of DMH acting on DNA with the same mechanism, several studies documented that* Mgmt* is upregulated by exposure to alkylating agents [11, 18]. Previous studies in rats also demonstrated that, shortly after AOM treatment, MGMT enzymatic activity is depleted and remains undetectable for two days [19]. Here we showed upregulation of* Mgmt* 24 h after DMH in wt rats. Given the suicidal mechanism of action of MGMT, our result is compatible with Nyskohus's findings [19] and suggests that the overexpression of the gene is due to a compensatory feedback mechanism. Although in DMH-treated Pirc rats the overexpression of* Mgmt* is not significant compared to that observed in DMH-treated wt rats, we document that the effect of DMH was present in both wt and Pirc rats, suggesting that the upregulation of* Mgmt* is a mechanism in common between the two genotypes.

GSTA1 and GSTP1 are members of the GST (glutathione S-transferase) superfamily of enzymes catalyzing the glutathione-dependent detoxification of electrophilic xenobiotics and certain products of oxidative stress [20]. Increased expression of GST isoforms has been associated with the beneficial effects of various dietary compounds* in vivo* [17, 21]. We found that in both genotypes* Gsta1* was significantly overexpressed in DMH-treated rats, indicating in both strains that this gene is inducible by DMH. At variance with* Gsta1*,* Gstp1* expression was not changed in both wt and Pirc rats after DMH; however, a significant effect of the genotype was observed, indicating that Pirc rats have a lower expression of this gene. It is interesting to note that* Min* mice mutated in* Apc* and null for* Gstp* gene showed enhanced intestinal carcinogenesis [22], suggesting a protective role for this gene. Therefore, it would be possible to speculate that the lower expression of* Gstp1* in Pirc rats could contribute to their spontaneous carcinogenesis as well as to their increased susceptibility to the carcinogenic effect of ethylnitrosourea [5], an alkylating agent which may also indirectly cause oxidative damage [23].

We previously showed that Pirc rats have a lower level of FRAP in the plasma when compared to wt rats [4]. Since this parameter may reflect the ability of plasma to counteract the toxic effect of oxidant xenobiotics, we thought that it was interesting to study its levels after DMH. Two-way ANOVA showed a significant effect of DMH in both genotypes; however, the analysis also highlighted a significant interaction between the two variables (genotype and DMH treatment) suggesting a different pattern of response to DMH in the two genotypes, which deserves further investigation.

To have a more comprehensive view of the responses to DMH beyond those predictable, we also performed whole genome expression profile analysis by using DNA microarray technology. Although previous studies evaluated variations in gene expression profile in cancer cells and animals in response to toxic treatments [15, 24], the response to genotoxic stress at whole gene level in animals mutated in* Apc* has not been studied so far.

The results of the unsupervised cluster analysis carried out on genes differentially affected in wt after DMH (fold change ≥ 3, *P* < 0.05) identified two clusters composed by genes that were overexpressed in wt rats but not changed in Pirc rats (21 genes) or downregulated in wt rats and overexpressed in Pirc rats (7 genes). Among these genes we noticed that some are regulated by IFN, namely,* Irf7*,* Oas1a*,* Oasl2*, and* Isg15*.* Irf7* is a member of the IFN regulatory factors (IRF), involved in defensive responses not only against viral infections but also against DNA-damaging chemicals [25, 26]. Accordingly, several chemotherapic drugs such as Adriamycin, Mitomycin, and Cisplatin activate Irf7 in cancer cell lines [25].* Oas1a* and* Oasl2* belong to the 2′-5′-oligoadenylate synthetase (OAS) family also induced by IFN and associated with antiviral and apoptotic response [26].* OAS* genes have been also associated with response to cytotoxic chemicals such as cyclophosphamide [27] and to radiations [24]. In agreement with these reports, we also found, at least in wt rats, upregulation of these genes after treatment with DMH. Verifying the array data with RT-PCR experiments, we found slight upregulation of these genes in DMH-treated Pirc rats. Analyzing data by two-way ANOVA, no significant differences between the two genotypes were found but only an effect of DMH, as also observed for* Mgmt* expression. The only exception was* Isg15*, a cytokine also induced by IFN [28], whose expression in both genotypes was not significantly varied after DMH, but two-way ANOVA analysis was shown to be significantly expressed at lower levels in Pirc rats.

In conclusion, the resistance to DMH-induced apoptosis in Pirc rats [4] is not mirrored by major differences at the whole genome level. However, a blunted induction of interferon-related genes (*Irf7* and* Oas1a*) and* Mgmt*, involved in the response to DMH, was observed. These changes, together with other minor differences, could explain the altered apoptotic response in Pirc rats.

## Supplementary Material

Supplementary Table 1, showing the list of the 101 genes significantly changed ( fold change (FC) of 3 or more compared to baseline), in wt rats after DMH-induction (p<0.05).
