# Supplementary material for: Gene Expression Profile of Colon Mucosa after Cytotoxic Insult in wt and Apc-Mutated Pirc Rats: Possible Relation to Resistance to Apoptosis during Carcinogenesis
Source: Biomed Res Int. 2016 Oct 20;2016:1310342. doi: 10.1155/2016/1310342 (PMC5093255; doi:10.1155/2016/1310342)
Supplement: Supplementary file 1 — Supplementary Table 1, showing the list of the 101 genes significantly changed ( fold change (FC) of 3 or more compared to baseline), in wt rats after DMH-induction (p<0.05). [file 1310342.f1.docx]

| **Supplementary Table 1**: List of the 101 genes significantly changed ( fold change (FC) of 3 or more compared to baseline), in wt rats after DMH-induction (p<0.05). For the same list of genes the FC observed in Pirc rats are also reported (Pirc-DMH vs Pirc). | | | | |
| --- | --- | --- | --- | --- |
| **ProbeName** | **Gene Name** | **Description** | **WT (FC)** | **Pirc (FC)** |
| A_64_P138859 | Pln | ref\|Rattus norvegicus phospholamban (Pln), mRNA [NM_022707] | 21.01 | 26.7 |
| A_64_P068960 | Hapln3 | ref\|Rattus norvegicus hyaluronan and proteoglycan link protein 3 (Hapln3), mRNA [NM_001008559] | 17.69 | 22.0 |
| A_64_P039888 | Abcb1b | ref\|Rattus norvegicus ATP-binding cassette, subfamily B (MDR/TAP), member 1B (Abcb1b), mRNA [NM_012623] | 7.57 | 14.9 |
| A_42_P624195 | Gsta3 | ref\|Rattus norvegicus glutathione S-transferase A3 (Gsta3), mRNA [NM_031509] | 6.43 | 13.6 |
| A_44_P503528 | Mptx | ref\|Rattus norvegicus mucosal pentraxin (Mptx), mRNA [NM_001037642] | -6.98 | 12.7 |
| A_64_P073829 | Lcn3 | ens\|Uncharacterized protein [Source:UniProtKB/TrEMBL;Acc:D3ZK46] [ENSRNOT00000061847] | 10.48 | 11.5 |
| A_44_P990376 | Gpnmb | ref\|Rattus norvegicus glycoprotein (transmembrane) nmb (Gpnmb), mRNA [NM_133298] | 10.17 | 10.8 |
| A_44_P1039128 | Cxcl10 | ref\|Rattus norvegicus chemokine (C-X-C motif) ligand 10 (Cxcl10), mRNA [NM_139089] | 8.72 | 9.2 |
| A_44_P288291 | Phlda3 | ref\|Rattus norvegicus pleckstrin homology-like domain, family A, member 3 (Phlda3), mRNA [NM_001012206] | 16.10 | 7.8 |
| A_64_P002546 | Retn | ref\|Rattus norvegicus resistin (Retn), mRNA [NM_144741] | -3.25 | 7.1 |
| A_44_P121417 | Pinlyp | ref\|Rattus norvegicus phospholipase A2 inhibitor and LY6/PLAUR domain containing (Pinlyp), mRNA [NM_001107488] | 9.65 | 7.0 |
| A_64_P100516 | Aoc3 | ref\|Rattus norvegicus amine oxidase, copper containing 3 (vascular adhesion protein 1) (Aoc3), mRNA [NM_031582] | -4.72 | 7.0 |
| A_43_P15750 | Cfd | ref\|Rattus norvegicus complement factor D (adipsin) (Cfd), mRNA [NM_001077642] | -4.44 | 6.7 |
| A_44_P360926 | Nlrp5 | ref\|Rattus norvegicus NLR family, pyrin domain containing 5 (Nlrp5), mRNA [NM_001107474] | 3.48 | 6.4 |
| A_44_P555271 | Mmp12 | ref\|Rattus norvegicus matrix metallopeptidase 12 (Mmp12), mRNA [NM_053963] | 4.87 | 5.6 |
| A_64_P059491 | Plcd4 | ref\|Rattus norvegicus phospholipase C, delta 4 (Plcd4), mRNA [NM_080688] | 3.13 | 5.3 |
| A_44_P1022458 | Tubb6 | ref\|Rattus norvegicus tubulin, beta 6 class V (Tubb6), mRNA [NM_001025675] | 3.44 | 5.1 |
| A_44_P244851 | Car3 | ref\|Rattus norvegicus carbonic anhydrase 3 (Car3), mRNA [NM_019292] | -7.83 | 4.6 |
| A_42_P538928 | Clu | ref\|Rattus norvegicus clusterin (Clu), mRNA [NM_053021] | 3.39 | 4.6 |
| A_64_P123118 | Fosl1 | ref\|Rattus norvegicus fos-like antigen 1 (Fosl1), mRNA [NM_012953] | 3.28 | 4.5 |
| A_64_P085530 | Scd1 | ref\|Rattus norvegicus stearoyl-Coenzyme A desaturase 1 (Scd1), mRNA [NM_139192] | -3.70 | 4.4 |
| A_44_P384017 | Sncg | ref\|Rattus norvegicus synuclein, gamma (breast cancer-specific protein 1) (Sncg), mRNA [NM_031688] | -4.17 | 4.2 |
| A_64_P016443 | Prkaca | ref\|Rattus norvegicus protein kinase, cAMP-dependent, catalytic, alpha (Prkaca), mRNA [NM_001100922] | 3.31 | 3.9 |
| A_42_P672071 | Ccl19 | ref\|Rattus norvegicus chemokine (C-C motif) ligand 19 (Ccl19), mRNA [NM_001108661] | -3.00 | 3.8 |
| A_64_P281091 | Rest | ref\|Rattus norvegicus RE1-silencing transcription factor (Rest), mRNA [NM_031788] | 3.76 | 3.8 |
| A_44_P367761 | Ccng1 | ref\|Rattus norvegicus cyclin G1 (Ccng1), mRNA [NM_012923] | 3.07 | 3.6 |
| A_64_P011489 | Gdf15 | ref\|Rattus norvegicus growth differentiation factor 15 (Gdf15), mRNA [NM_019216] | 3.87 | 3.5 |
| A_64_P042694 | Gtlf3b | ref\|Rattus norvegicus gene trap locus F3b (Gtlf3b), mRNA [NM_001170541] | 3.69 | 3.5 |
| A_44_P187056 | Itga6 | ref\|Rattus norvegicus integrin, alpha 6 (Itga6), mRNA [NM_053725] | 4.34 | 3.3 |
| A_43_P11590 | Mmp7 | ref\|Rattus norvegicus matrix metallopeptidase 7 (Mmp7), mRNA [NM_012864] | 4.47 | 3.3 |
| A_64_P001824 | Slc12a6 | ref\|Rattus norvegicus solute carrier family 12, member 6 (Slc12a6), mRNA [NM_001109630] | 3.05 | 3.2 |
| A_44_P541297 | Slc27a3 | ref\|Rattus norvegicus solute carrier family 27 (fatty acid transporter), member 3 (Slc27a3), mRNA [NM_001106439] | 3.62 | 3.2 |
| A_44_P218284 | Dusp3 | ref\|Rattus norvegicus dual specificity phosphatase 3 (Dusp3), mRNA [NM_001173376] | 3.18 | 3.1 |
| A_42_P485932 | Sulf2 | ref\|Rattus norvegicus sulfatase 2 (Sulf2), mRNA [NM_001034927] | 3.52 | 3.1 |
| A_64_P141727 | Rhob | ref\|Rattus norvegicus ras homolog family member B (Rhob), mRNA [NM_022542] | 3.44 | 3.0 |
| A_64_P159330 | Adam8 | ens\|Uncharacterized protein [Source:UniProtKB/TrEMBL;Acc:D3ZB52] [ENSRNOT00000024369] | 3.24 | 2.9 |
| A_44_P352268 | Plk2 | ref\|Rattus norvegicus polo-like kinase 2 (Plk2), mRNA [NM_031821] | 3.16 | 2.6 |
| A_64_P024416 | Parp14 | ref\|Rattus norvegicus poly (ADP-ribose) polymerase family, member 14 (Parp14), mRNA [NM_001191659] | 4.04 | 2.5 |
| A_64_P074870 | Elf4 | ref\|Rattus norvegicus E74-like factor 4 (ets domain transcription factor) (Elf4), mRNA [NM_001191735] | 3.53 | 2.4 |
| A_44_P185355 | Bcl11b | ref\|Rattus norvegicus B-cell CLL/lymphoma 11B (zinc finger protein) (Bcl11b), mRNA [NM_001108057] | 3.43 | 2.3 |
| A_44_P159584 | Med15 | ref\|Rattus norvegicus mediator complex subunit 15 (Med15), mRNA [NM_001108325] | 3.01 | 2.2 |
| A_64_P078002 | Triobp | ref\|Rattus norvegicus TRIO and F-actin binding protein (Triobp), mRNA [NM_001013220] | 3.10 | 2.0 |
| A_42_P470966 | Msi2 | ref\|PREDICTED: Rattus norvegicus Musashi homolog 2 (Drosophila) (Msi2), mRNA [XM_340876] | 3.32 | 2.0 |
| A_44_P199368 | Kdm4a | ref\|Rattus norvegicus lysine (K)-specific demethylase 4A (Kdm4a), mRNA [NM_001107966] | 3.26 | 2.0 |
| A_44_P255341 | Ipo9 | ref\|Rattus norvegicus importin 9 (Ipo9), mRNA [NM_001107180] | 3.22 | 2.0 |
| A_44_P351211 | Phlda1 | ref\|Rattus norvegicus pleckstrin homology-like domain, family A, member 1 (Phlda1), mRNA [NM_017180] | 5.06 | 1.9 |
| A_64_P016525 | Ffar3 | ref\|Rattus norvegicus free fatty acid receptor 3 (Ffar3), mRNA [NM_001108912] | 3.34 | 1.9 |
| A_44_P865962 | Mapre3 | ref\|Rattus norvegicus microtubule-associated protein, RP/EB family, member 3 (Mapre3), mRNA [NM_001007656] | 3.12 | 1.7 |
| A_64_P043161 | Fbln1 | ref\|Rattus norvegicus fibulin 1 (Fbln1), mRNA [NM_001127547] | 3.29 | 1.6 |
| A_43_P21195 | Dll4 | ref\|Rattus norvegicus delta-like 4 (Drosophila) (Dll4), mRNA [NM_001107760] | 3.01 | 1.6 |
| A_64_P137996 | Zfp444 | ref\|Rattus norvegicus zinc finger protein 444 (Zfp444), mRNA [NM_001169143] | 3.10 | 1.6 |
| A_64_P075928 | Foxe1 | ref\|Rattus norvegicus forkhead box E1 (thyroid transcription factor 2) (Foxe1), mRNA [NM_138909] | 3.10 | 1.5 |
| A_64_P148413 | Ces1d | ref\|Rattus norvegicus carboxylesterase 1D (Ces1d), mRNA [NM_133295] | -6.30 | 1.4 |
| A_44_P381917 | Sphk1 | ref\|Rattus norvegicus sphingosine kinase 1 (Sphk1), transcript variant 6, mRNA [NM_133386] | 3.56 | 1.4 |
| A_44_P1045748 | Lpin2 | ref\|Rattus norvegicus lipin 2 (Lpin2), mRNA [NM_001108236] | 4.00 | 1.4 |
| A_42_P559831 | Ces1c | ref\|Rattus norvegicus carboxylesterase 1C (Ces1c), mRNA [NM_017004] | -3.42 | 1.4 |
| A_44_P126061 | Oasl2 | ref\|Rattus norvegicus 2'-5' oligoadenylate synthetase-like 2 (Oasl2), mRNA [NM_001009682] | 4.47 | 1.4 |
| A_44_P486312 | Fmo2 | ref\|Rattus norvegicus flavin containing monooxygenase 2 (Fmo2), mRNA [NM_144737] | -3.10 | 1.3 |
| A_43_P16471 | Taf6l | ref\|Rattus norvegicus TAF6-like RNA polymerase II, p300/CBP-associated factor (PCAF)-associated factor (Taf6l), mRNA [NM_001107575] | 3.18 | 1.3 |
| A_64_P058615 | Uncx | ref\|Rattus norvegicus UNC homeobox (Uncx), mRNA [NM_017179] | -3.35 | 1.3 |
| A_64_P021806 | Kpna6 | ref\|Rattus norvegicus karyopherin alpha 6 (importin alpha 7) (Kpna6), mRNA [NM_001015029] | 3.42 | 1.2 |
| A_44_P1013314 | Isg15 | ref\|Rattus norvegicus ISG15 ubiquitin-like modifier (Isg15), mRNA [NM_001106700] | 4.79 | 1.2 |
| A_44_P382312 | Pric285 | ens\|Uncharacterized protein [Source:UniProtKB/TrEMBL;Acc:D3ZFS4] [ENSRNOT00000017787] | 3.11 | 1.2 |
| A_44_P1028196 | Ly6e | ref\|Rattus norvegicus lymphocyte antigen 6 complex, locus E (Ly6e), mRNA [NM_001017467] | 3.70 | 1.1 |
| A_64_P157455 | Eid3 | ref\|Rattus norvegicus EP300 interacting inhibitor of differentiation 3 (Eid3), mRNA [NM_001044304] | -3.13 | 1.1 |
| A_64_P017473 | Chmp4bl1 | ref\|PREDICTED: Rattus norvegicus chromatin modifying protein 4B-like 1 (Chmp4bl1), mRNA [XM_003749717] | 3.87 | 1.1 |
| A_64_P070821 | Neto1 | ref\|Rattus norvegicus neuropilin (NRP) and tolloid (TLL)-like 1 (Neto1), mRNA [NM_001107371] | -3.11 | 1.0 |
| A_64_P041219 | Pcsk1 | ref\|Rattus norvegicus proprotein convertase subtilisin/kexin type 1 (Pcsk1), mRNA [NM_017091] | -3.22 | 1.0 |
| A_44_P359169 | Prr11 | ref\|Rattus norvegicus proline rich 11 (Prr11), mRNA [NM_001108287] | -3.00 | 1.0 |
| A_64_P019331 | Orai2 | ref\|Rattus norvegicus ORAI calcium release-activated calcium modulator 2 (Orai2), mRNA [NM_001170403] | 3.07 | 1.0 |
| A_44_P1039994 | Irf7 | ref\|Rattus norvegicus interferon regulatory factor 7 (Irf7), mRNA [NM_001033691] | 3.12 | -1.0 |
| A_43_P20512 | Lzts2 | ref\|Rattus norvegicus leucine zipper, putative tumor suppressor 2 (Lzts2), mRNA [NM_001014247] | 3.55 | -1.1 |
| A_42_P787410 | Cacna1b | ref\|Rattus norvegicus calcium channel, voltage-dependent, N type, alpha 1B subunit (Cacna1b), transcript variant 2, mRNA [NM_147141] | -3.25 | -1.1 |
| A_64_P124701 | Crlf1 | ref\|Rattus norvegicus cytokine receptor-like factor 1 (Crlf1), mRNA [NM_001106074] | -3.69 | -1.2 |
| A_44_P313542 | Sgk1 | ref\|Rattus norvegicus serum/glucocorticoid regulated kinase 1 (Sgk1), transcript variant 3, mRNA [NM_019232] | 3.28 | -1.2 |
| A_64_P150338 | Tmem132c | ens\|Uncharacterized protein [Source:UniProtKB/TrEMBL;Acc:F1LYB3] [ENSRNOT00000048474] | -3.14 | -1.2 |
| A_64_P038567 | Herc6 | ens\|Uncharacterized protein [Source:UniProtKB/TrEMBL;Acc:D3ZT29] [ENSRNOT00000058626] | 3.65 | -1.2 |
| A_64_P040130 | Casc4 | ref\|Rattus norvegicus cancer susceptibility candidate 4 (Casc4), mRNA [NM_001271283] | -3.20 | -1.3 |
| A_42_P481087 | Pdzrn3 | ref\|Rattus norvegicus PDZ domain containing RING finger 3 (Pdzrn3), mRNA [NM_001271251] | -4.16 | -1.4 |
| A_64_P057606 | Abl1 | ref\|Rattus norvegicus c-abl oncogene 1, non-receptor tyrosine kinase (Abl1), mRNA [NM_001100850] | 3.36 | -1.4 |
| A_42_P812805 | Smoc2 | ref\|Rattus norvegicus SPARC related modular calcium binding 2 (Smoc2), mRNA [NM_001106215] | -3.02 | -1.4 |
| A_42_P766165 | Afap1l1 | ref\|Rattus norvegicus actin filament associated protein 1-like 1 (Afap1l1), mRNA [NM_001106142] | -3.94 | -1.4 |
| A_64_P139500 | Vom1r38 | ref\|Rattus norvegicus vomeronasal 1 receptor 38 (Vom1r38), mRNA [NM_001008964] | -3.19 | -1.5 |
| A_44_P992662 | Ftcd | ref\|Rattus norvegicus formiminotransferase cyclodeaminase (Ftcd), mRNA [NM_053567] | -3.11 | -1.6 |
| A_64_P013324 | Tcf7l1 | ref\|Rattus norvegicus transcription factor 7-like 1 (T-cell specific, HMG-box) (Tcf7l1), mRNA [NM_001107865] | -3.43 | -1.6 |
| A_44_P487468 | Olfm4 | ref\|Rattus norvegicus olfactomedin 4 (Olfm4), mRNA [NM_001106052] | -3.37 | -1.7 |
| A_64_P025790 | Oas1a | ref\|Rattus norvegicus 2'-5' oligoadenylate synthetase 1A (Oas1a), mRNA [NM_138913] | 3.88 | -1.7 |
| A_64_P147338 | Chrna10 | ref\|Rattus norvegicus cholinergic receptor, nicotinic, alpha 10 (neuronal) (Chrna10), mRNA [NM_022639] | -3.29 | -1.7 |
| A_64_P025839 | Oas1k | ref\|Rattus norvegicus 2 ' -5 ' oligoadenylate synthetase 1K (Oas1k), mRNA [NM_001009489] | 3.89 | -1.8 |
| A_44_P1051894 | Tmem132c | ref\|PREDICTED: Rattus norvegicus transmembrane protein 132C (Tmem132c), mRNA [XM_002724836] | -3.98 | -1.9 |
| A_64_P011779 | Sult1a1 | ref\|Rattus norvegicus sulfotransferase family, cytosolic, 1A, phenol-preferring, member 1 (Sult1a1), mRNA [NM_031834] | -4.27 | -1.9 |
| A_44_P140514 | Cenpa | ref\|Rattus norvegicus centromere protein A (Cenpa), mRNA [NM_001106711] | -3.04 | -1.9 |
| A_64_P159722 | Bub1 | ref\|Rattus norvegicus budding uninhibited by benzimidazoles 1 homolog (S. cerevisiae) (Bub1), mRNA [NM_001106507] | -3.46 | -2.0 |
| A_64_P032585 | Hist1h2an | ref\|Rattus norvegicus histone cluster 1, H2an (Hist1h2an), mRNA [NM_001107354] | -3.99 | -2.0 |
| A_64_P165536 | Bpifb6 | ref\|Rattus norvegicus BPI fold containing family B, member 6 (Bpifb6), mRNA [NM_001107791] | -3.48 | -2.0 |
| A_64_P158653 | Knstrn | ref\|Rattus norvegicus kinetochore-localized astrin/SPAG5 binding protein (Knstrn), mRNA [NM_001004264] | -3.00 | -2.0 |
| A_44_P454532 | Ambp | ref\|Rattus norvegicus alpha-1-microglobulin/bikunin precursor (Ambp), mRNA [NM_012901] | -3.06 | -2.1 |
| A_64_P020238 | Hist1h2bf | ref\|PREDICTED: Rattus norvegicus histone cluster 1, H2bf (Hist1h2bf), mRNA [XM_001061682] | -3.03 | -2.4 |
| A_42_P567268 | Mt2A | ref\|Rattus norvegicus metallothionein 2A (Mt2A), mRNA [NM_001137564] | -3.80 | -2.7 |
| A_44_P219972 | Nt5e | ref\|Rattus norvegicus 5' nucleotidase, ecto (Nt5e), mRNA [NM_021576] | -3.34 | -3.5 |
| A_64_P021043 | Pcsk9 | ref\|Rattus norvegicus proprotein convertase subtilisin/kexin type 9 (Pcsk9), mRNA [NM_199253] | -18.87 | -6.2 |

| **Supplementary Table 2**: List of the 432 genes with fold change (FC) of 3 or more, compared to baseline, in Pirc rats after DMH-induction. For the same list of genes the FC observed in wt rats are also reported (wt-DMH vs wt). | | | | |
| --- | --- | --- | --- | --- |
| **ProbeName** | **Gene Name** | **Description** | **Pirc (FC)** | **WT (FC)** |
| A_64_P138859 | Pln | ref\|Rattus norvegicus phospholamban (Pln), mRNA [NM_022707] | 26.74 | 21.01 |
| A_64_P068960 | Hapln3 | ref\|Rattus norvegicus hyaluronan and proteoglycan link protein 3 (Hapln3), mRNA [NM_001008559] | 22.03 | 17.69 |
| A_64_P039888 | Abcb1b | ref\|Rattus norvegicus ATP-binding cassette, subfamily B (MDR/TAP), member 1B (Abcb1b), mRNA [NM_012623] | 14.92 | 7.57 |
| A_42_P624195 | Gsta3 | ref\|Rattus norvegicus glutathione S-transferase A3 (Gsta3), mRNA [NM_031509] | 13.60 | 6.43 |
| A_44_P360767 | Spink3 | ref\|Rattus norvegicus serine peptidase inhibitor, Kazal type 3 (Spink3), mRNA [NM_012674] | 13.52 | 1.92 |
| A_44_P503528 | Mptx | ref\|Rattus norvegicus mucosal pentraxin (Mptx), mRNA [NM_001037642] | 12.69 | -6.98 |
| A_64_P073829 | Lcn3 | ens\|Uncharacterized protein [Source:UniProtKB/TrEMBL;Acc:D3ZK46] [ENSRNOT00000061847] | 11.48 | 10.48 |
| A_44_P990376 | Gpnmb | ref\|Rattus norvegicus glycoprotein (transmembrane) nmb (Gpnmb), mRNA [NM_133298] | 10.83 | 10.17 |
| A_64_P048526 | S1pr1 | ref\|Rattus norvegicus sphingosine-1-phosphate receptor 1 (S1pr1), mRNA [NM_017301] | 9.32 | 1.44 |
| A_44_P1039128 | Cxcl10 | ref\|Rattus norvegicus chemokine (C-X-C motif) ligand 10 (Cxcl10), mRNA [NM_139089] | 9.20 | 8.72 |
| A_64_P078332 | Glycam1 | ref\|Rattus norvegicus glycosylation dependent cell adhesion molecule 1 (Glycam1), mRNA [NM_012794] | 8.99 | -1.82 |
| A_64_P018181 | Retnlb | ref\|Rattus norvegicus resistin like beta (Retnlb), mRNA [NM_001024281] | 8.85 | -1.84 |
| A_64_P070155 | Rhox5 | ref\|Rattus norvegicus reproductive homeobox 5 (Rhox5), mRNA [NM_022175] | 7.89 | -1.95 |
| A_44_P288291 | Phlda3 | ref\|Rattus norvegicus pleckstrin homology-like domain, family A, member 3 (Phlda3), mRNA [NM_001012206] | 7.84 | 16.10 |
| A_42_P535644 | Adamts1 | ref\|Rattus norvegicus ADAM metallopeptidase with thrombospondin type 1 motif, 1 (Adamts1), mRNA [NM_024400] | 7.58 | 1.17 |
| A_64_P069256 | Ccr6 | ref\|Rattus norvegicus chemokine (C-C motif) receptor 6 (Ccr6), mRNA [NM_001013145] | 7.43 | 1.96 |
| A_43_P10102 | Spry4 | ref\|Rattus norvegicus sprouty homolog 4 (Drosophila) (Spry4), mRNA [NM_001106150] | 7.42 | 1.70 |
| A_43_P12772 | Retnla | ref\|Rattus norvegicus resistin like alpha (Retnla), mRNA [NM_053333] | 7.35 | 1.12 |
| A_64_P150607 | Defb40 | ref\|Rattus norvegicus defensin beta 40 (Defb40), mRNA [NM_001037511] | 7.11 | -1.45 |
| A_64_P002546 | Retn | ref\|Rattus norvegicus resistin (Retn), mRNA [NM_144741] | 7.07 | -3.25 |
| A_44_P121417 | Pinlyp | ref\|Rattus norvegicus phospholipase A2 inhibitor and LY6/PLAUR domain containing (Pinlyp), mRNA [NM_001107488] | 7.02 | 9.65 |
| A_64_P100516 | Aoc3 | ref\|Rattus norvegicus amine oxidase, copper containing 3 (vascular adhesion protein 1) (Aoc3), mRNA [NM_031582] | 7.00 | -4.72 |
| A_43_P15750 | Cfd | ref\|Rattus norvegicus complement factor D (adipsin) (Cfd), mRNA [NM_001077642] | 6.72 | -4.44 |
| A_64_P135464 | Tff1 | ref\|Rattus norvegicus trefoil factor 1 (Tff1), mRNA [NM_057129] | 6.52 | 2.64 |
| A_44_P360926 | Nlrp5 | ref\|Rattus norvegicus NLR family, pyrin domain containing 5 (Nlrp5), mRNA [NM_001107474] | 6.36 | 3.48 |
| A_64_P088382 | Angptl4 | ref\|Rattus norvegicus angiopoietin-like 4 (Angptl4), mRNA [NM_199115] | 6.18 | 1.21 |
| A_43_P17088 | Agpat5 | ref\|Rattus norvegicus 1-acylglycerol-3-phosphate O-acyltransferase 5 (lysophosphatidic acid acyltransferase, epsilon) (Agpat5), mRNA [NM_001134744] | 6.16 | 1.48 |
| A_64_P013437 | Cxcl13 | ref\|Rattus norvegicus chemokine (C-X-C motif) ligand 13 (Cxcl13), mRNA [NM_001017496] | 6.04 | -1.25 |
| A_64_P008723 | Sval1 | ref\|Rattus norvegicus seminal vesicle antigen-like 1 (Sval1), mRNA [NM_133292] | 5.76 | 1.54 |
| A_64_P080434 | Hoxb7 | ref\|Rattus norvegicus homeo box B7 (Hoxb7), mRNA [NM_001017480] | 5.68 | -1.28 |
| A_44_P555271 | Mmp12 | ref\|Rattus norvegicus matrix metallopeptidase 12 (Mmp12), mRNA [NM_053963] | 5.59 | 4.87 |
| A_44_P271658 | Reg3b | ref\|Rattus norvegicus regenerating islet-derived 3 beta (Reg3b), mRNA [NM_053289] | 5.50 | 1.71 |
| A_64_P099821 | Prl2a1 | ref\|Rattus norvegicus Prolactin family 2, subfamily a, member 1 (Prl2a1), mRNA [NM_053791] | 5.41 | 2.59 |
| A_43_P12786 | Fabp4 | ref\|Rattus norvegicus fatty acid binding protein 4, adipocyte (Fabp4), mRNA [NM_053365] | 5.40 | -1.35 |
| A_64_P128549 | Bicc1 | ref\|Rattus norvegicus bicaudal C homolog 1 (Drosophila) (Bicc1), mRNA [NM_001108531] | 5.37 | -1.07 |
| A_43_P12743 | Atp7a | ref\|Rattus norvegicus ATPase, Cu++ transporting, alpha polypeptide (Atp7a), mRNA [NM_052803] | 5.35 | 2.06 |
| A_43_P13127 | Slc38a4 | ref\|Rattus norvegicus solute carrier family 38, member 4 (Slc38a4), mRNA [NM_130748] | 5.33 | 1.60 |
| A_64_P059491 | Plcd4 | ref\|Rattus norvegicus phospholipase C, delta 4 (Plcd4), mRNA [NM_080688] | 5.27 | 3.13 |
| A_44_P531758 | Cryab | ref\|Rattus norvegicus crystallin, alpha B (Cryab), mRNA [NM_012935] | 5.18 | 1.37 |
| A_64_P083349 | Kitlg | ref\|Rattus norvegicus KIT ligand (Kitlg), transcript variant 1, mRNA [NM_021843] | 5.11 | 1.57 |
| A_44_P1022458 | Tubb6 | ref\|Rattus norvegicus tubulin, beta 6 class V (Tubb6), mRNA [NM_001025675] | 5.10 | 3.44 |
| A_44_P353618 | S100a9 | ref\|Rattus norvegicus S100 calcium binding protein A9 (S100a9), mRNA [NM_053587] | 5.10 | 1.52 |
| A_64_P099900 | Kap | ref\|Rattus norvegicus kidney androgen regulated protein (Kap), mRNA [NM_052802] | 5.06 | 2.75 |
| A_44_P382501 | Foxr1 | ens\|Uncharacterized protein [Source:UniProtKB/TrEMBL;Acc:F1LTW6] [ENSRNOT00000049125] | 5.02 | -2.59 |
| A_44_P1055780 | S100a8 | ref\|Rattus norvegicus S100 calcium binding protein A8 (S100a8), mRNA [NM_053822] | 5.01 | 1.29 |
| A_43_P16715 | Sertad2 | ref\|Rattus norvegicus SERTA domain containing 2 (Sertad2), mRNA [NM_001024903] | 4.97 | 1.95 |
| A_44_P943756 | Ccdc82 | ref\|Rattus norvegicus coiled-coil domain containing 82 (Ccdc82), mRNA [NM_001007660] | 4.96 | 1.57 |
| A_44_P125733 | Madcam1 | ref\|Rattus norvegicus mucosal vascular addressin cell adhesion molecule 1 (Madcam1), mRNA [NM_019317] | 4.80 | 1.02 |
| A_44_P714839 | Txlnb | ref\|Rattus norvegicus taxilin beta (Txlnb), mRNA [NM_001135859] | 4.80 | 1.20 |
| A_42_P499158 | Vcam1 | ref\|Rattus norvegicus vascular cell adhesion molecule 1 (Vcam1), mRNA [NM_012889] | 4.79 | -1.19 |
| A_43_P10083 | Rgma | ref\|Rattus norvegicus RGM domain family, member A (Rgma), mRNA [NM_001107524] | 4.77 | 1.01 |
| A_44_P1030225 | Apoa4 | ref\|Rattus norvegicus apolipoprotein A-IV (Apoa4), mRNA [NM_012737] | 4.71 | 2.77 |
| A_64_P114258 | Alg10 | ref\|Rattus norvegicus asparagine-linked glycosylation 10, alpha-1,2-glucosyltransferase homolog (S. pombe) (Alg10), mRNA [NM_139101] | 4.68 | 1.59 |
| A_44_P244851 | Car3 | ref\|Rattus norvegicus carbonic anhydrase 3 (Car3), mRNA [NM_019292] | 4.61 | -7.83 |
| A_42_P538928 | Clu | ref\|Rattus norvegicus clusterin (Clu), mRNA [NM_053021] | 4.60 | 3.39 |
| A_44_P991940 | Socs6 | ref\|Rattus norvegicus suppressor of cytokine signaling 6 (Socs6), mRNA [NM_001271149] | 4.60 | -1.08 |
| A_64_P123118 | Fosl1 | ref\|Rattus norvegicus fos-like antigen 1 (Fosl1), mRNA [NM_012953] | 4.48 | 3.28 |
| A_44_P440178 | Lhfp | ref\|Rattus norvegicus lipoma HMGIC fusion partner (Lhfp), mRNA [NM_001109183] | 4.47 | 1.48 |
| A_64_P100318 | Mar-01 | ref\|Rattus norvegicus mitochondrial amidoxime reducing component 1 (Marc1), nuclear gene encoding mitochondrial protein, mRNA [NM_001100811] | 4.46 | 1.24 |
| A_44_P306307 | Hbb | ref\|Rattus norvegicus hemoglobin, beta (Hbb), mRNA [NM_033234] | 4.42 | 1.37 |
| A_64_P054376 | Gpr4 | ref\|Rattus norvegicus G protein-coupled receptor 4 (Gpr4), mRNA [NM_001025680] | 4.42 | 1.32 |
| A_44_P193945 | Bst1 | ref\|Rattus norvegicus bone marrow stromal cell antigen 1 (Bst1), mRNA [NM_030848] | 4.42 | 2.36 |
| A_64_P085530 | Scd1 | ref\|Rattus norvegicus stearoyl-Coenzyme A desaturase 1 (Scd1), mRNA [NM_139192] | 4.38 | -3.70 |
| A_64_P082678 | Nkd1 | ref\|Rattus norvegicus naked cuticle homolog 1 (Drosophila) (Nkd1), mRNA [NM_001271381] | 4.37 | 1.82 |
| A_64_P151259 | C2cd4a | ref\|PREDICTED: Rattus norvegicus C2 calcium-dependent domain containing 4A (C2cd4a), mRNA [XM_001079329] | 4.35 | 2.35 |
| A_64_P287740 | Rpgr | ens\|retinitis pigmentosa GTPase regulator [Source:RefSeq peptide;Acc:NP_001121073] [ENSRNOT00000067603] | 4.35 | -1.24 |
| A_64_P028406 | Rprd1b | ref\|Rattus norvegicus regulation of nuclear pre-mRNA domain containing 1B (Rprd1b), mRNA [NM_001098727] | 4.35 | 1.94 |
| A_44_P928825 | Msr1 | ref\|Rattus norvegicus macrophage scavenger receptor 1 (Msr1), mRNA [NM_001191939] | 4.32 | 1.00 |
| A_42_P538268 | Chsy1 | ref\|Rattus norvegicus chondroitin sulfate synthase 1 (Chsy1), mRNA [NM_001106268] | 4.30 | 1.40 |
| A_64_P024949 | Xcl1 | ref\|Rattus norvegicus chemokine (C motif) ligand 1 (Xcl1), mRNA [NM_134361] | 4.28 | 2.02 |
| A_44_P447650 | Adarb1 | ref\|Rattus norvegicus adenosine deaminase, RNA-specific, B1 (Adarb1), transcript variant 1, mRNA [NM_012894] | 4.25 | 2.75 |
| A_44_P540992 | Ereg | ref\|Rattus norvegicus epiregulin (Ereg), mRNA [NM_021689] | 4.23 | 2.99 |
| A_44_P384017 | Sncg | ref\|Rattus norvegicus synuclein, gamma (breast cancer-specific protein 1) (Sncg), mRNA [NM_031688] | 4.20 | -4.17 |
| A_64_P035852 | Lcorl | ref\|PREDICTED: Rattus norvegicus similar to Mblk1-related protein-1 (RGD1561241), mRNA [XM_001058603] | 4.19 | 1.05 |
| A_64_P148508 | Prr7 | ref\|Rattus norvegicus proline rich 7 (synaptic) (Prr7), mRNA [NM_001109116] | 4.10 | 1.79 |
| A_44_P105069 | Rab32 | ref\|Rattus norvegicus RAB32, member RAS oncogene family (Rab32), mRNA [NM_001108902] | 4.10 | 2.20 |
| A_64_P155320 | Sval1 | ref\|Rattus norvegicus seminal vesicle antigen-like 1 (Sval1), mRNA [NM_133292] | 4.08 | 1.42 |
| A_42_P825912 | Slc39a10 | ref\|PREDICTED: Rattus norvegicus solute carrier family 39 (zinc transporter), member 10 (Slc39a10), mRNA [XM_003750698] | 4.07 | 2.18 |
| A_42_P824217 | Dapp1 | ref\|Rattus norvegicus dual adaptor of phosphotyrosine and 3-phosphoinositides (Dapp1), mRNA [NM_001108568] | 4.05 | 1.05 |
| A_64_P152790 | Frzb | ref\|Rattus norvegicus frizzled-related protein (Frzb), mRNA [NM_001100527] | 4.05 | -1.01 |
| A_44_P325508 | Anxa3 | ref\|Rattus norvegicus annexin A3 (Anxa3), mRNA [NM_012823] | 4.04 | 2.16 |
| A_44_P321075 | Rasal2 | ref\|Rattus norvegicus RAS protein activator like 2 (Rasal2), mRNA [NM_001107188] | 4.03 | 2.48 |
| A_64_P159034 | Nat8l | ref\|Rattus norvegicus N-acetyltransferase 8-like (Nat8l), mRNA [NM_001191681] | 3.95 | -2.06 |
| A_64_P147204 | Ahnak | ref\|Rattus norvegicus AHNAK nucleoprotein (Ahnak), mRNA [NM_001191951] | 3.94 | 4.15 |
| A_64_P016443 | Prkaca | ref\|Rattus norvegicus protein kinase, cAMP-dependent, catalytic, alpha (Prkaca), mRNA [NM_001100922] | 3.90 | 3.31 |
| A_64_P118753 | Ccnl1 | ref\|Rattus norvegicus cyclin L1 (Ccnl1), mRNA [NM_053662] | 3.89 | 1.12 |
| A_44_P435596 | Zfp36 | ref\|Rattus norvegicus zinc finger protein 36 (Zfp36), mRNA [NM_133290] | 3.88 | 1.41 |
| A_42_P780457 | Enc1 | ref\|Rattus norvegicus ectodermal-neural cortex 1 (Enc1), mRNA [NM_001003401] | 3.87 | 2.92 |
| A_64_P006898 | Cd19 | ref\|Rattus norvegicus CD19 molecule (Cd19), mRNA [NM_001013237] | 3.85 | -1.56 |
| A_64_P005857 | Gpr18 | ref\|Rattus norvegicus G protein-coupled receptor 18 (Gpr18), mRNA [NM_001079710] | 3.84 | 1.04 |
| A_44_P237994 | Abcg1 | ref\|Rattus norvegicus ATP-binding cassette, subfamily G (WHITE), member 1 (Abcg1), mRNA [NM_053502] | 3.84 | 1.42 |
| A_42_P672071 | Ccl19 | ref\|Rattus norvegicus chemokine (C-C motif) ligand 19 (Ccl19), mRNA [NM_001108661] | 3.84 | -3.00 |
| A_64_P113793 | Cd36 | ref\|Rattus norvegicus CD36 molecule (thrombospondin receptor) (Cd36), mRNA [NM_031561] | 3.82 | 1.00 |
| A_64_P281091 | Rest | ref\|Rattus norvegicus RE1-silencing transcription factor (Rest), mRNA [NM_031788] | 3.81 | 3.76 |
| A_44_P1009603 | Gtse1 | ref\|Rattus norvegicus G-2 and S-phase expressed 1 (Gtse1), mRNA [NM_001130500] | 3.80 | 1.16 |
| A_44_P530801 | Mcpt8l2 | ref\|Rattus norvegicus mast cell protease 8-like 2 (Mcpt8l2), mRNA [NM_001135010] | 3.75 | 1.07 |
| A_64_P109754 | Epgn | ens\|Uncharacterized protein [Source:UniProtKB/TrEMBL;Acc:D3Z9P6] [ENSRNOT00000003720] | 3.75 | 2.32 |
| A_44_P140148 | Cd248 | ref\|Rattus norvegicus CD248 molecule, endosialin (Cd248), mRNA [NM_001106325] | 3.75 | -1.02 |
| A_44_P445070 | Lgals1 | ref\|Rattus norvegicus lectin, galactoside-binding, soluble, 1 (Lgals1), mRNA [NM_019904] | 3.72 | 1.05 |
| A_64_P009633 | Tmprss7 | ref\|Rattus norvegicus transmembrane protease, serine 7 (Tmprss7), mRNA [NM_001105882] | 3.69 | 2.42 |
| A_64_P081391 | Hoxd10 | ref\|Rattus norvegicus homeo box D10 (Hoxd10), mRNA [NM_001107094] | 3.68 | -2.31 |
| A_64_P164804 | Gpr18 | ref\|Rattus norvegicus G protein-coupled receptor 18 (Gpr18), mRNA [NM_001079710] | 3.67 | 1.44 |
| A_64_P150519 | Drd4 | ref\|Rattus norvegicus dopamine receptor D4 (Drd4), mRNA [NM_012944] | 3.66 | 1.53 |
| A_43_P12374 | Lpar3 | ref\|Rattus norvegicus lysophosphatidic acid receptor 3 (Lpar3), mRNA [NM_023969] | 3.65 | 2.80 |
| A_44_P540660 | Ccdc50 | ref\|Rattus norvegicus coiled-coil domain containing 50 (Ccdc50), mRNA [NM_182736] | 3.65 | 1.67 |
| A_44_P964460 | Itk | ref\|Rattus norvegicus IL2-inducible T-cell kinase (Itk), mRNA [NM_001108825] | 3.64 | 1.35 |
| A_64_P001542 | Rps27l | ref\|PREDICTED: Rattus norvegicus ribosomal protein S27-like (Rps27l), mRNA [XM_001056747] | 3.63 | 1.82 |
| A_64_P100013 | Prrc2c | ens\|Uncharacterized protein [Source:UniProtKB/TrEMBL;Acc:D3ZGV8] [ENSRNOT00000004410] | 3.59 | 1.32 |
| A_64_P044806 | Zfp28 | ref\|PREDICTED: Rattus norvegicus zinc finger protein 758 (Zfp758), mRNA [XM_001074805] | 3.58 | 1.22 |
| A_44_P367761 | Ccng1 | ref\|Rattus norvegicus cyclin G1 (Ccng1), mRNA [NM_012923] | 3.57 | 3.07 |
| A_64_P112816 | Gen1 | ref\|Rattus norvegicus Gen endonuclease homolog 1 (Drosophila) (Gen1), mRNA [NM_001106717] | 3.57 | 1.47 |
| A_42_P804822 | Scn7a | ref\|Rattus norvegicus sodium channel, voltage-gated, type VII, alpha (Scn7a), mRNA [NM_031686] | 3.56 | 1.10 |
| A_44_P471901 | Ptbp3 | ref\|Rattus norvegicus polypyrimidine tract binding protein 3 (Ptbp3), mRNA [NM_031346] | 3.55 | 1.65 |
| A_64_P019160 | Etnk1 | ref\|Rattus norvegicus ethanolamine kinase 1 (Etnk1), mRNA [NM_001107894] | 3.55 | 1.83 |
| A_44_P364447 | Spats2l | ref\|Rattus norvegicus spermatogenesis associated, serine-rich 2-like (Spats2l), mRNA [NM_001014102] | 3.55 | 2.35 |
| A_64_P058953 | Defb43 | ref\|Rattus norvegicus defensin beta 43 (Defb43), mRNA [NM_001037528] | 3.55 | -1.89 |
| A_64_P069216 | Gramd1c | ref\|Rattus norvegicus GRAM domain containing 1C (Gramd1c), mRNA [NM_001191826] | 3.53 | 1.42 |
| A_64_P116301 | Adam33 | ref\|PREDICTED: Rattus norvegicus ADAM metallopeptidase domain 33 (Adam33), mRNA [XM_003749574] | 3.53 | -1.40 |
| A_44_P682817 | Myo15 | ens\|Uncharacterized protein [Source:UniProtKB/TrEMBL;Acc:D3ZJY7] [ENSRNOT00000023018] | 3.50 | -1.15 |
| A_64_P150904 | Rgcc | ref\|Rattus norvegicus regulator of cell cycle (Rgcc), mRNA [NM_054008] | 3.50 | -1.65 |
| A_64_P011489 | Gdf15 | ref\|Rattus norvegicus growth differentiation factor 15 (Gdf15), mRNA [NM_019216] | 3.50 | 3.87 |
| A_64_P024655 | Aebp1 | ref\|Rattus norvegicus AE binding protein 1 (Aebp1), mRNA [NM_001100970] | 3.48 | -1.04 |
| A_64_P042694 | Gtlf3b | ref\|Rattus norvegicus gene trap locus F3b (Gtlf3b), mRNA [NM_001170541] | 3.48 | 3.69 |
| A_64_P054808 | Cd36 | ref\|Rattus norvegicus CD36 molecule (thrombospondin receptor) (Cd36), mRNA [NM_031561] | 3.47 | -1.19 |
| A_64_P112579 | Slc16a11 | ref\|Rattus norvegicus solute carrier family 16, member 11 (monocarboxylic acid transporter 11) (Slc16a11), mRNA [NM_001105797] | 3.46 | 1.38 |
| A_42_P821316 | Elf5 | ref\|Rattus norvegicus E74-like factor 5 (Elf5), mRNA [NM_001108956] | 3.46 | 1.11 |
| A_42_P551104 | Ccdc80 | ref\|Rattus norvegicus coiled-coil domain containing 80 (Ccdc80), mRNA [NM_022543] | 3.45 | -2.03 |
| A_42_P707818 | Tmcc2 | ens\|Similar to RIKEN cDNA 1110063G11 (Predicted)Uncharacterized protein [Source:UniProtKB/TrEMBL;Acc:D3ZE26] [ENSRNOT00000000036] | 3.45 | 1.63 |
| A_42_P723641 | Popdc2 | ref\|Rattus norvegicus popeye domain containing 2 (Popdc2), mRNA [NM_199113] | 3.44 | 2.15 |
| A_42_P473594 | Egr2 | ref\|Rattus norvegicus early growth response 2 (Egr2), mRNA [NM_053633] | 3.44 | 1.98 |
| A_42_P723244 | Arhgef4 | ens\|Similar to Rho guanine nucleotide exchange factor 4 isoform a, isoform CRA_aUncharacterized protein [Source:UniProtKB/TrEMBL;Acc:D3ZKB4] [ENSRNOT00000018935] | 3.44 | 1.65 |
| A_64_P125676 | Sptbn4 | ref\|Rattus norvegicus spectrin, beta, non-erythrocytic 4 (Sptbn4), mRNA [NM_001100845] | 3.43 | 2.29 |
| A_64_P007618 | Gfra3 | ref\|Rattus norvegicus GDNF family receptor alpha 3 (Gfra3), mRNA [NM_053398] | 3.42 | 1.17 |
| A_43_P15530 | S100b | ref\|Rattus norvegicus S100 calcium binding protein B (S100b), mRNA [NM_013191] | 3.41 | -1.54 |
| A_44_P303883 | Tbx2 | ref\|Rattus norvegicus T-box 2 (Tbx2), mRNA [NM_001107033] | 3.40 | -1.16 |
| A_64_P154927 | Mylk | ref\|Rattus norvegicus myosin light chain kinase (Mylk), mRNA [NM_001105874] | 3.39 | 2.96 |
| A_64_P159110 | Aqpep | ens\|Uncharacterized protein [Source:UniProtKB/TrEMBL;Acc:D3ZZW8] [ENSRNOT00000039733] | 3.39 | -1.02 |
| A_44_P870348 | Slc35d3 | ref\|Rattus norvegicus solute carrier family 35, member D3 (Slc35d3), mRNA [NM_001107522] | 3.39 | 1.30 |
| A_64_P017791 | Skor1 | ref\|PREDICTED: Rattus norvegicus SKI family transcriptional corepressor 1, transcript variant 3 (Skor1), mRNA [XM_002727063] | 3.39 | 1.43 |
| A_44_P154138 | Rxfp2 | ref\|Rattus norvegicus relaxin/insulin-like family peptide receptor 2 (Rxfp2), mRNA [NM_001012475] | 3.39 | -1.16 |
| A_44_P285575 | Cox6c-ps1 | ref\|Rattus norvegicus cytochrome c oxidase subunit VIc, pseudogene (Cox6c-ps1), non-coding RNA [NR_037621] | 3.39 | 1.12 |
| A_44_P335079 | Tff3 | ref\|Rattus norvegicus trefoil factor 3, intestinal (Tff3), mRNA [NM_013042] | 3.38 | 2.28 |
| A_64_P012057 | Atp2b1 | ref\|Rattus norvegicus ATPase, Ca++ transporting, plasma membrane 1 (Atp2b1), mRNA [NM_053311] | 3.38 | 2.98 |
| A_44_P548303 | Thumpd1 | ref\|Rattus norvegicus THUMP domain containing 1 (Thumpd1), mRNA [NM_001009688] | 3.37 | -1.03 |
| A_64_P085660 | Nipal3 | ref\|PREDICTED: Rattus norvegicus NIPA-like domain containing 3 (Nipal3), mRNA [XM_003754133] | 3.37 | -1.03 |
| A_64_P067613 | Pgr | ref\|Rattus norvegicus progesterone receptor (Pgr), mRNA [NM_022847] | 3.37 | 1.46 |
| A_42_P696084 | Cspg4 | ref\|Rattus norvegicus chondroitin sulfate proteoglycan 4 (Cspg4), mRNA [NM_031022] | 3.37 | -1.43 |
| A_64_P096773 | Gdf3 | ref\|Rattus norvegicus growth differentiation factor 3 (Gdf3), mRNA [NM_001109671] | 3.37 | 1.94 |
| A_64_P005832 | Ggact | ref\|Rattus norvegicus gamma-glutamylamine cyclotransferase (Ggact), mRNA [NM_001025634] | 3.35 | 1.44 |
| A_64_P119438 | Pramef12 | ref\|PREDICTED: Rattus norvegicus PRAME family member 12 (Pramef12), mRNA [XM_001077034] | 3.34 | 1.90 |
| A_42_P647599 | Dcn | ref\|Rattus norvegicus decorin (Dcn), mRNA [NM_024129] | 3.33 | -1.03 |
| A_64_P058087 | Rbp4 | ref\|Rattus norvegicus retinol binding protein 4, plasma (Rbp4), mRNA [NM_013162] | 3.33 | -2.33 |
| A_64_P037196 | Nacc1 | ref\|Rattus norvegicus nucleus accumbens associated 1, BEN and BTB (POZ) domain containing (Nacc1), mRNA [NM_134413] | 3.33 | 1.80 |
| A_44_P653272 | Gpr155 | ref\|Rattus norvegicus G protein-coupled receptor 155 (Gpr155), mRNA [NM_001107811] | 3.32 | -1.30 |
| A_44_P187056 | Itga6 | ref\|Rattus norvegicus integrin, alpha 6 (Itga6), mRNA [NM_053725] | 3.32 | 4.34 |
| A_42_P833106 | Rad54l2 | ref\|Rattus norvegicus Rad54 like 2 (S. cerevisiae) (Rad54l2), mRNA [NM_001134520] | 3.32 | 1.96 |
| A_44_P124733 | Zp2 | ref\|Rattus norvegicus zona pellucida glycoprotein 2 (sperm receptor) (Zp2), mRNA [NM_031150] | 3.32 | 2.07 |
| A_64_P079751 | Bst1 | ref\|Rattus norvegicus bone marrow stromal cell antigen 1 (Bst1), mRNA [NM_030848] | 3.31 | 1.41 |
| A_44_P429453 | Lama5 | ref\|Rattus norvegicus laminin, alpha 5 (Lama5), mRNA [NM_001191609] | 3.29 | 2.40 |
| A_64_P004231 | Gsta5 | ref\|Rattus norvegicus glutathione S-transferase Yc2 subunit (Gsta5), transcript variant 2, mRNA [NM_001159739] | 3.29 | 2.35 |
| A_44_P300618 | Usp24 | ref\|Rattus norvegicus ubiquitin specific peptidase 24 (Usp24), mRNA [NM_001271206] | 3.27 | 1.76 |
| A_42_P460021 | Adcy10 | ref\|Rattus norvegicus adenylate cyclase 10 (soluble) (Adcy10), mRNA [NM_021684] | 3.27 | 1.37 |
| A_64_P032162 | Gldc | ref\|Rattus norvegicus glycine dehydrogenase (decarboxylating) (Gldc), nuclear gene encoding mitochondrial protein, mRNA [NM_001107583] | 3.26 | 1.44 |
| A_64_P029112 | Pvrl1 | ens\|Uncharacterized protein [Source:UniProtKB/TrEMBL;Acc:F1LNP8] [ENSRNOT00000008614] | 3.26 | 1.05 |
| A_64_P091953 | Spink4 | ref\|Rattus norvegicus serine peptidase inhibitor, Kazal type 4 (Spink4), mRNA [NM_001008871] | 3.26 | 1.92 |
| A_43_P11590 | Mmp7 | ref\|Rattus norvegicus matrix metallopeptidase 7 (Mmp7), mRNA [NM_012864] | 3.25 | 4.47 |
| A_44_P427596 | Fabp1 | ref\|Rattus norvegicus fatty acid binding protein 1, liver (Fabp1), mRNA [NM_012556] | 3.25 | 1.70 |
| A_64_P057456 | Ptprcap | ref\|Rattus norvegicus protein tyrosine phosphatase, receptor type, C-associated protein (Ptprcap), mRNA [NM_001024289] | 3.24 | 1.71 |
| A_64_P109327 | S100b | ref\|Rattus norvegicus S100 calcium binding protein B (S100b), mRNA [NM_013191] | 3.24 | -3.34 |
| A_64_P001824 | Slc12a6 | ref\|Rattus norvegicus solute carrier family 12, member 6 (Slc12a6), mRNA [NM_001109630] | 3.22 | 3.05 |
| A_44_P914961 | Otud7b | ref\|Rattus norvegicus OTU domain containing 7B (Otud7b), mRNA [NM_001107697] | 3.22 | 2.46 |
| A_44_P466595 | Eif4e3 | ref\|Rattus norvegicus eukaryotic translation initiation factor 4E family member 3 (Eif4e3), mRNA [NM_001106612] | 3.22 | 2.64 |
| A_64_P008234 | Zfp580 | ref\|PREDICTED: Rattus norvegicus zinc finger protein 580, transcript variant 1 (Zfp580), mRNA [XM_001072400] | 3.21 | 1.90 |
| A_44_P366723 | Igf1 | ref\|Rattus norvegicus insulin-like growth factor 1 (Igf1), transcript variant 2, mRNA [NM_178866] | 3.20 | -1.11 |
| A_44_P1043157 | Cxcl9 | ref\|Rattus norvegicus chemokine (C-X-C motif) ligand 9 (Cxcl9), mRNA [NM_145672] | 3.19 | 2.02 |
| A_64_P031691 | Actg2 | ref\|Rattus norvegicus actin, gamma 2, smooth muscle, enteric (Actg2), mRNA [NM_012893] | 3.18 | 1.44 |
| A_44_P233080 | Egr1 | ens\|Early growth response protein 1 [Source:UniProtKB/Swiss-Prot;Acc:P08154] [ENSRNOT00000026303] | 3.18 | 1.78 |
| A_64_P139173 | Cd40 | ref\|Rattus norvegicus CD40 molecule, TNF receptor superfamily member 5 (Cd40), mRNA [NM_134360] | 3.18 | 2.14 |
| A_43_P11672 | Il1rl1 | ref\|Rattus norvegicus interleukin 1 receptor-like 1 (Il1rl1), transcript variant 1, mRNA [NM_013037] | 3.17 | 1.63 |
| A_64_P132511 | Mcc | ref\|Rattus norvegicus mutated in colorectal cancers (Mcc), mRNA [NM_001170534] | 3.17 | -1.68 |
| A_43_P11484 | Mme | ref\|Rattus norvegicus membrane metallo-endopeptidase (Mme), mRNA [NM_012608] | 3.17 | 1.40 |
| A_43_P12767 | Pdlim5 | ref\|Rattus norvegicus PDZ and LIM domain 5 (Pdlim5), mRNA [NM_053326] | 3.17 | 2.04 |
| A_44_P541297 | Slc27a3 | ref\|Rattus norvegicus solute carrier family 27 (fatty acid transporter), member 3 (Slc27a3), mRNA [NM_001106439] | 3.15 | 3.62 |
| A_44_P458021 | Tnfrsf11b | ref\|Rattus norvegicus tumor necrosis factor receptor superfamily, member 11b (Tnfrsf11b), mRNA [NM_012870] | 3.15 | 1.73 |
| A_64_P152888 | Nphp4 | ref\|Rattus norvegicus nephronophthisis 4 (juvenile) homolog (human) (Nphp4), mRNA [NM_001037650] | 3.15 | 1.30 |
| A_44_P522847 | Pik3ap1 | ref\|Rattus norvegicus phosphoinositide-3-kinase adaptor protein 1 (Pik3ap1), mRNA [NM_001106368] | 3.15 | -1.16 |
| A_64_P144913 | Nab2 | ref\|Rattus norvegicus Ngfi-A binding protein 2 (Nab2), mRNA [NM_001134874] | 3.14 | 1.88 |
| A_44_P218284 | Dusp3 | ref\|Rattus norvegicus dual specificity phosphatase 3 (Dusp3), mRNA [NM_001173376] | 3.14 | 3.18 |
| A_44_P100197 | Zfp799 | ref\|Rattus norvegicus zinc finger protein 799 (Zfp799), mRNA [NM_001009537] | 3.14 | 2.03 |
| A_64_P116259 | Cpsf2 | ref\|Rattus norvegicus cleavage and polyadenylation specific factor 2, 100kDa (Cpsf2), mRNA [NM_001106753] | 3.14 | 1.73 |
| A_64_P166118 | Rax | ref\|Rattus norvegicus retina and anterior neural fold homeobox (Rax), mRNA [NM_053678] | 3.14 | 2.34 |
| A_42_P485932 | Sulf2 | ref\|Rattus norvegicus sulfatase 2 (Sulf2), mRNA [NM_001034927] | 3.14 | 3.52 |
| A_44_P389034 | Tp53inp2 | ref\|Rattus norvegicus tumor protein p53 inducible nuclear protein 2 (Tp53inp2), mRNA [NM_001270947] | 3.14 | 1.60 |
| A_64_P022210 | Il20 | ref\|Rattus norvegicus interleukin 20 (Il20), mRNA [NM_001143881] | 3.13 | -1.00 |
| A_64_P024356 | Sh2b2 | ref\|Rattus norvegicus SH2B adaptor protein 2 (Sh2b2), mRNA [NM_053669] | 3.13 | -1.27 |
| A_64_P048013 | Arhgap28 | ref\|Rattus norvegicus Rho GTPase activating protein 28 (Arhgap28), mRNA [NM_001191815] | 3.12 | 2.37 |
| A_64_P002250 | Adh6a | ref\|Rattus norvegicus alcohol dehydrogenase 6A (class V) (Adh6a), mRNA [NM_001106475] | 3.11 | -1.27 |
| A_44_P496327 | Prf1 | ref\|Rattus norvegicus perforin 1 (pore forming protein) (Prf1), mRNA [NM_017330] | 3.10 | 2.59 |
| A_44_P885445 | Nxpe3 | ref\|Rattus norvegicus neurexophilin and PC-esterase domain family, member 3 (Nxpe3), mRNA [NM_001109435] | 3.10 | -1.22 |
| A_64_P071495 | Actg2 | ref\|Rattus norvegicus actin, gamma 2, smooth muscle, enteric (Actg2), mRNA [NM_012893] | 3.10 | 1.36 |
| A_64_P080429 | Hoxb6 | ref\|PREDICTED: Rattus norvegicus homeo box B6 (Hoxb6), mRNA [XM_573182] | 3.09 | -1.18 |
| A_44_P239306 | Ydjc | ref\|Rattus norvegicus YdjC homolog (bacterial) (Ydjc), mRNA [NM_001013863] | 3.08 | 1.02 |
| A_44_P764174 | Faim3 | ref\|Rattus norvegicus Fas apoptotic inhibitory molecule 3 (Faim3), mRNA [NM_001014843] | 3.08 | 1.26 |
| A_42_P828695 | Gpx8 | ref\|Rattus norvegicus glutathione peroxidase 8 (Gpx8), mRNA [NM_001106411] | 3.08 | 1.54 |
| A_44_P550662 | Ms4a6a | ens\|Uncharacterized protein [Source:UniProtKB/TrEMBL;Acc:D3ZZ58] [ENSRNOT00000032937] | 3.07 | 2.08 |
| A_42_P508984 | Pf4 | ref\|Rattus norvegicus platelet factor 4 (Pf4), mRNA [NM_001007729] | 3.06 | 1.29 |
| A_43_P11534 | Klkb1 | ref\|Rattus norvegicus kallikrein B, plasma 1 (Klkb1), mRNA [NM_012725] | 3.06 | -1.47 |
| A_64_P027288 | Timd4 | ref\|PREDICTED: Rattus norvegicus T-cell immunoglobulin and mucin domain-containing protein 4-like (RGD1564516), mRNA [XM_002724488] | 3.05 | -1.07 |
| A_44_P345081 | Cenpm | ref\|Rattus norvegicus centromere protein M (Cenpm), mRNA [NM_001130504] | 3.05 | 1.46 |
| A_44_P118724 | Arc | ref\|Rattus norvegicus activity-regulated cytoskeleton-associated protein (Arc), mRNA [NM_019361] | 3.04 | 1.73 |
| A_64_P156198 | Olr903 | ref\|Rattus norvegicus olfactory receptor 903 (Olr903), mRNA [NM_001001361] | 3.04 | 1.28 |
| A_64_P138919 | Eln | ref\|Rattus norvegicus elastin (Eln), mRNA [NM_012722] | 3.04 | -1.39 |
| A_43_P20980 | Tacc1 | ref\|Rattus norvegicus transforming, acidic coiled-coil containing protein 1 (Tacc1), mRNA [NM_001004107] | 3.04 | -1.76 |
| A_42_P585995 | Nim1 | ref\|PREDICTED: Rattus norvegicus serine/threonine-protein kinase NIM1 (Nim1), mRNA [XM_001076547] | 3.03 | 1.84 |
| A_64_P056698 | Pom121l2 | ref\|Rattus norvegicus POM121 transmembrane nucleoporin-like 2 (Pom121l2), transcript variant 2, mRNA [NM_001162931] | 3.03 | 1.15 |
| A_44_P396040 | Tmem2 | ref\|Rattus norvegicus transmembrane protein 2 (Tmem2), mRNA [NM_001107596] | 3.03 | 2.91 |
| A_44_P352538 | Sectm1 | ref\|Rattus norvegicus secreted and transmembrane 1 (Sectm1), mRNA [NM_001013043] | 3.02 | 1.29 |
| A_64_P033924 | Atf6 | ref\|Rattus norvegicus activating transcription factor 6 (Atf6), mRNA [NM_001107196] | 3.02 | 1.28 |
| A_44_P435422 | Mef2a | ref\|Rattus norvegicus myocyte enhancer factor 2a (Mef2a), mRNA [NM_001014035] | 3.02 | 2.87 |
| A_44_P382482 | Atp6v1c2 | ref\|Rattus norvegicus ATPase, H+ transporting, lysosomal V1 subunit C2 (Atp6v1c2), mRNA [NM_001014199] | 3.01 | 1.66 |
| A_64_P121946 | Psapl1 | ref\|Rattus norvegicus prosaposin-like 1 (Psapl1), mRNA [NM_001144850] | 3.01 | 3.68 |
| A_44_P668373 | Vasn | ref\|Rattus norvegicus vasorin (Vasn), mRNA [NM_001109382] | 3.00 | -1.22 |
| A_44_P945807 | Fbxo10 | ref\|PREDICTED: Rattus norvegicus F-box protein 10 (Fbxo10), mRNA [XM_001071167] | 3.00 | 1.49 |
| A_43_P15544 | Ltb4r | ref\|Rattus norvegicus leukotriene B4 receptor (Ltb4r), mRNA [NM_021656] | -3.00 | -1.49 |
| A_64_P097138 | Zfp219 | ref\|Rattus norvegicus zinc finger protein 219 (Zfp219), mRNA [NM_001007681] | -3.01 | 1.87 |
| A_44_P325782 | Hsd3b7 | ref\|Rattus norvegicus hydroxy-delta-5-steroid dehydrogenase, 3 beta- and steroid delta-isomerase 7 (Hsd3b7), mRNA [NM_139329] | -3.01 | 1.76 |
| A_64_P120734 | Egf | ref\|Rattus norvegicus epidermal growth factor (Egf), mRNA [NM_012842] | -3.02 | -2.37 |
| A_64_P115157 | Ces1e | ref\|Rattus norvegicus carboxylesterase 1E (Ces1e), mRNA [NM_031565] | -3.02 | -2.56 |
| A_43_P10642 | Poldip3 | ref\|Rattus norvegicus polymerase (DNA-directed), delta interacting protein 3 (Poldip3), mRNA [NM_001130506] | -3.02 | -1.13 |
| A_44_P465233 | Fgfbp1 | ref\|Rattus norvegicus fibroblast growth factor binding protein 1 (Fgfbp1), mRNA [NM_022603] | -3.03 | -1.95 |
| A_44_P309097 | Sctr | ref\|Rattus norvegicus secretin receptor (Sctr), mRNA [NM_031115] | -3.03 | -1.69 |
| A_64_P103610 | Lmod3 | ens\|Uncharacterized protein [Source:UniProtKB/TrEMBL;Acc:D4A871] [ENSRNOT00000047453] | -3.04 | -2.14 |
| A_44_P339572 | Olr257 | ref\|Rattus norvegicus olfactory receptor 257 (Olr257), mRNA [NM_001001038] | -3.04 | -1.33 |
| A_44_P447158 | Pdlim3 | ens\|PDZ and LIM domain protein 3 [Source:UniProtKB/Swiss-Prot;Acc:Q66HS7] [ENSRNOT00000017568] | -3.04 | -2.09 |
| A_44_P405139 | Arhgef11 | ref\|Rattus norvegicus Rho guanine nucleotide exchange factor (GEF) 11 (Arhgef11), mRNA [NM_023982] | -3.07 | 1.41 |
| A_44_P154493 | Gstt4 | ref\|Rattus norvegicus glutathione S-transferase, theta 4 (Gstt4), mRNA [NM_001109675] | -3.07 | -1.31 |
| A_64_P054900 | Pcdhb7 | ref\|PREDICTED: Rattus norvegicus protocadherin beta 7 (Pcdhb7), mRNA [XM_001055294] | -3.09 | -1.14 |
| A_44_P543157 | Olr1213 | ref\|Rattus norvegicus olfactory receptor 1213 (Olr1213), mRNA [NM_001000815] | -3.09 | -1.31 |
| A_64_P072763 | Olr454 | ref\|Rattus norvegicus olfactory receptor 454 (Olr454), mRNA [NM_001000290] | -3.10 | 1.13 |
| A_64_P063398 | Ptger3 | ref\|Rattus norvegicus prostaglandin E receptor 3 (subtype EP3) (Ptger3), mRNA [NM_012704] | -3.10 | -2.25 |
| A_44_P414421 | Olr1087 | ref\|Rattus norvegicus olfactory receptor 1087 (Olr1087), mRNA [NM_001000418] | -3.11 | -2.12 |
| A_64_P026523 | Olr1265 | ref\|Rattus norvegicus olfactory receptor 1265 (Olr1265), mRNA [NM_001000457] | -3.11 | -1.21 |
| A_44_P208049 | Sh2b1 | ref\|Rattus norvegicus SH2B adaptor protein 1 (Sh2b1), transcript variant 1, mRNA [NM_134456] | -3.11 | 1.95 |
| A_44_P436336 | Mageh1 | ref\|Rattus norvegicus melanoma antigen, family H, 1 (Mageh1), mRNA [NM_001013250] | -3.12 | -1.26 |
| A_44_P438090 | Plcb3 | ref\|Rattus norvegicus phospholipase C, beta 3 (phosphatidylinositol-specific) (Plcb3), mRNA [NM_033350] | -3.12 | -1.26 |
| A_43_P12591 | Oprl1 | ref\|Rattus norvegicus opiate receptor-like 1 (Oprl1), mRNA [NM_031569] | -3.12 | -1.13 |
| A_64_P071858 | Zfp641 | ref\|Rattus norvegicus zinc finger protein 641 (Zfp641), mRNA [NM_001106792] | -3.13 | -1.23 |
| A_44_P193130 | Sftpa1 | ref\|Rattus norvegicus surfactant protein A1 (Sftpa1), transcript variant 2, mRNA [NM_017329] | -3.13 | -1.59 |
| A_64_P065081 | Ihh | ref\|Rattus norvegicus Indian hedgehog (Ihh), mRNA [NM_053384] | -3.14 | 1.95 |
| A_64_P037587 | Vom2r36 | ref\|Rattus norvegicus vomeronasal 2 receptor, 36 (Vom2r36), mRNA [NM_001099483] | -3.14 | 1.20 |
| A_64_P023227 | Abcg3l4 | ref\|Rattus norvegicus ATP-binding cassette, subfamily G (WHITE), member 3-like 4 (Abcg3l4), mRNA [NM_001037205] | -3.15 | -1.53 |
| A_44_P454331 | Olr1086 | ref\|Rattus norvegicus olfactory receptor 1086 (Olr1086), mRNA [NM_001000419] | -3.15 | -1.24 |
| A_44_P178179 | Pgbd5 | ref\|Rattus norvegicus piggyBac transposable element derived 5 (Pgbd5), mRNA [NM_001106198] | -3.15 | -1.18 |
| A_64_P058794 | Olr853 | ref\|Rattus norvegicus olfactory receptor 853 (Olr853), mRNA [NM_001000398] | -3.15 | -1.55 |
| A_44_P503570 | Parg | ref\|Rattus norvegicus poly (ADP-ribose) glycohydrolase (Parg), mRNA [NM_031339] | -3.15 | -1.08 |
| A_42_P803406 | Nags | ref\|Rattus norvegicus N-acetylglutamate synthase (Nags), nuclear gene encoding mitochondrial protein, mRNA [NM_001107053] | -3.15 | 1.06 |
| A_64_P130699 | Fgf11 | ref\|Rattus norvegicus fibroblast growth factor 11 (Fgf11), mRNA [NM_130816] | -3.15 | -1.33 |
| A_44_P520441 | Pcdh9 | ref\|Rattus norvegicus protocadherin 9 (Pcdh9), mRNA [NM_001191688] | -3.16 | -1.01 |
| A_44_P806532 | Dnai1 | ref\|Rattus norvegicus dynein, axonemal, intermediate chain 1 (Dnai1), mRNA [NM_001024342] | -3.16 | -2.38 |
| A_44_P218305 | Igsf11 | ref\|Rattus norvegicus immunoglobulin superfamily, member 11 (Igsf11), mRNA [NM_001013120] | -3.17 | -1.11 |
| A_64_P090229 | Olr824 | ref\|Rattus norvegicus olfactory receptor 824 (Olr824), mRNA [NM_001000905] | -3.17 | -1.07 |
| A_64_P138636 | Nr2f1 | ref\|Rattus norvegicus nuclear receptor subfamily 2, group F, member 1 (Nr2f1), mRNA [NM_031130] | -3.17 | 1.36 |
| A_64_P024424 | Kera | ref\|Rattus norvegicus keratocan (Kera), mRNA [NM_001108087] | -3.18 | -1.86 |
| A_64_P028773 | Zfp787 | ref\|Rattus norvegicus zinc finger protein 787 (Zfp787), mRNA [NM_001108904] | -3.18 | 2.18 |
| A_64_P085645 | Rpl10l | ref\|PREDICTED: Rattus norvegicus ribosomal protein L10-like (Rpl10l), mRNA [XM_003750173] | -3.19 | -1.29 |
| A_64_P120108 | RT1-CE12 | ref\|Rattus norvegicus RT1 class I, locus CE12 (RT1-CE12), mRNA [NM_001008835] | -3.19 | 2.40 |
| A_43_P23200 | Ptges2 | ref\|Rattus norvegicus prostaglandin E synthase 2 (Ptges2), mRNA [NM_001107832] | -3.20 | 1.65 |
| A_42_P542727 | Ano4 | ref\|Rattus norvegicus anoctamin 4 (Ano4), mRNA [NM_001106778] | -3.20 | -2.76 |
| A_64_P079967 | Spaca1 | ref\|Rattus norvegicus sperm acrosome associated 1 (Spaca1), mRNA [NM_001191982] | -3.21 | 1.00 |
| A_64_P155523 | Olr415 | ref\|Rattus norvegicus olfactory receptor 415 (Olr415), mRNA [NM_001000386] | -3.22 | 1.00 |
| A_42_P756334 | Myh6 | ref\|Rattus norvegicus myosin, heavy chain 6, cardiac muscle, alpha (Myh6), mRNA [NM_017239] | -3.23 | -1.16 |
| A_44_P156589 | Ngef | ref\|Rattus norvegicus neuronal guanine nucleotide exchange factor (Ngef), mRNA [NM_001136241] | -3.23 | 1.38 |
| A_64_P078376 | Olr404 | ref\|Rattus norvegicus olfactory receptor 404 (Olr404), mRNA [NM_001000382] | -3.23 | -1.09 |
| A_64_P056341 | Olr1485 | ref\|Rattus norvegicus olfactory receptor 1485 (Olr1485), mRNA [NM_001000028] | -3.24 | 1.16 |
| A_43_P18397 | Tfap2b | ref\|Rattus norvegicus transcription factor AP-2 beta (Tfap2b), mRNA [NM_001106896] | -3.24 | 1.04 |
| A_64_P098931 | Ahdc1 | ref\|Rattus norvegicus AT hook, DNA binding motif, containing 1 (Ahdc1), mRNA [NM_001134956] | -3.25 | -1.07 |
| A_64_P158525 | Egfem1 | ref\|Rattus norvegicus EGF-like and EMI domain containing 1 (Egfem1), mRNA [NM_001107663] | -3.26 | -2.17 |
| A_44_P389462 | Zmym3 | ref\|Rattus norvegicus zinc finger, MYM-type 3 (Zmym3), mRNA [NM_001040155] | -3.26 | -1.07 |
| A_44_P471201 | Mybl1 | ref\|Rattus norvegicus myeloblastosis oncogene-like 1 (Mybl1), mRNA [NM_001106632] | -3.26 | -2.53 |
| A_44_P473047 | Nos1 | ref\|Rattus norvegicus nitric oxide synthase 1, neuronal (Nos1), mRNA [NM_052799] | -3.28 | 1.08 |
| A_44_P128007 | Slco2b1 | ref\|Rattus norvegicus solute carrier organic anion transporter family, member 2b1 (Slco2b1), mRNA [NM_080786] | -3.28 | -1.39 |
| A_64_P043956 | Glp2r | ref\|Rattus norvegicus glucagon-like peptide 2 receptor (Glp2r), mRNA [NM_021848] | -3.31 | -1.66 |
| A_64_P097884 | Svs1 | ref\|Rattus norvegicus seminal vesicle secretory protein 1 (Svs1), mRNA [NM_199095] | -3.32 | -2.84 |
| A_64_P148265 | Ttll11 | ens\|Uncharacterized protein [Source:UniProtKB/TrEMBL;Acc:F1LPS9] [ENSRNOT00000032204] | -3.34 | -1.45 |
| A_42_P688442 | Usf1 | ref\|Rattus norvegicus upstream transcription factor 1 (Usf1), mRNA [NM_031777] | -3.35 | 1.04 |
| A_44_P170396 | Timm44 | ref\|Rattus norvegicus translocase of inner mitochondrial membrane 44 homolog (yeast) (Timm44), nuclear gene encoding mitochondrial protein, mRNA [NM_017267] | -3.35 | -1.47 |
| A_64_P093561 | Olr6 | ref\|Rattus norvegicus olfactory receptor 6 (Olr6), mRNA [NM_001000539] | -3.37 | 1.04 |
| A_64_P103060 | Olr1330 | ref\|Rattus norvegicus olfactory receptor 1330 (Olr1330), mRNA [NM_001000476] | -3.38 | -1.08 |
| A_44_P444257 | Cdkn2d | ref\|Rattus norvegicus similar to cyclin-dependent kinase inhibitor 2D (Cdkn2d), mRNA [NM_001009719] | -3.39 | -1.79 |
| A_64_P073859 | Crtc3 | ens\|Uncharacterized protein [Source:UniProtKB/TrEMBL;Acc:F1LVL6] [ENSRNOT00000015948] | -3.40 | -1.04 |
| A_64_P117251 | Tll2 | ref\|Rattus norvegicus tolloid-like 2 (Tll2), mRNA [NM_001191898] | -3.40 | -1.88 |
| A_44_P147055 | Gjb1 | ref\|Rattus norvegicus gap junction protein, beta 1 (Gjb1), mRNA [NM_017251] | -3.40 | -1.12 |
| A_64_P148852 | Agap2 | ref\|Rattus norvegicus ArfGAP with GTPase domain, ankyrin repeat and PH domain 2 (Agap2), mRNA [NM_023026] | -3.41 | -1.40 |
| A_64_P006498 | Ces1e | ref\|Rattus norvegicus carboxylesterase 1E (Ces1e), mRNA [NM_031565] | -3.41 | -2.20 |
| A_64_P026577 | Usf2 | ref\|Rattus norvegicus upstream transcription factor 2, c-fos interacting (Usf2), mRNA [NM_031139] | -3.42 | 1.43 |
| A_64_P112305 | Rpl39l | ref\|Rattus norvegicus similar to RIKEN cDNA 4930517K11 (LOC497860), mRNA [NM_001195471] | -3.42 | -4.97 |
| A_44_P482254 | Olr1421 | ref\|Rattus norvegicus olfactory receptor 1421 (Olr1421), mRNA [NM_001000780] | -3.43 | -2.94 |
| A_64_P092772 | Atp13a5 | ref\|Rattus norvegicus ATPase type 13A5 (Atp13a5), mRNA [NM_001191657] | -3.44 | -1.55 |
| A_64_P089025 | Ier5 | ref\|Rattus norvegicus immediate early response 5 (Ier5), mRNA [NM_001025137] | -3.47 | 1.99 |
| A_64_P393176 | Ces2j | ref\|Rattus norvegicus carboxylesterase 2J (Ces2j), mRNA [NM_001190380] | -3.47 | -1.64 |
| A_64_P045907 | Vom2r22 | ref\|Rattus norvegicus vomeronasal 2 receptor, 22 (Vom2r22), mRNA [NM_001099656] | -3.47 | 1.00 |
| A_64_P052825 | Fcer1a | ref\|Rattus norvegicus Fc fragment of IgE, high affinity I, receptor for; alpha polypeptide (Fcer1a), mRNA [NM_012724] | -3.47 | 1.00 |
| A_64_P107737 | Tnfrsf13c | ref\|PREDICTED: Rattus norvegicus tumor necrosis factor receptor superfamily, member 13c (Tnfrsf13c), mRNA [XM_001077542] | -3.47 | -1.70 |
| A_43_P12385 | Zfp354c | ref\|Rattus norvegicus zinc finger protein 354C (Zfp354c), mRNA [NM_023988] | -3.47 | 1.32 |
| A_44_P219972 | Nt5e | ref\|Rattus norvegicus 5' nucleotidase, ecto (Nt5e), mRNA [NM_021576] | -3.47 | -3.34 |
| A_64_P153062 | Tspan9 | ref\|Rattus norvegicus tetraspanin 9 (Tspan9), mRNA [NM_001107890] | -3.49 | -1.11 |
| A_44_P478298 | Agap3 | ref\|Rattus norvegicus ArfGAP with GTPase domain, ankyrin repeat and PH domain 3 (Agap3), mRNA [NM_001108616] | -3.50 | 1.73 |
| A_42_P662710 | Dedd | ref\|Rattus norvegicus death effector domain-containing (Dedd), mRNA [NM_031800] | -3.50 | 1.64 |
| A_44_P294914 | Sec1 | ref\|Rattus norvegicus secretory blood group 1 (Sec1), mRNA [NM_001135584] | -3.50 | -1.27 |
| A_64_P016097 | Dhrsx | ref\|Rattus norvegicus dehydrogenase/reductase (SDR family) X chromosome (Dhrsx), mRNA [NM_001105914] | -3.51 | -1.25 |
| A_44_P684199 | Dhrs7 | ref\|Rattus norvegicus dehydrogenase/reductase (SDR family) member 7 (Dhrs7), mRNA [NM_001013098] | -3.53 | -1.41 |
| A_64_P052680 | Dnmt1 | ref\|Rattus norvegicus DNA (cytosine-5-)-methyltransferase 1 (Dnmt1), mRNA [NM_053354] | -3.53 | -1.40 |
| A_64_P048334 | Vom2r38 | ref\|Rattus norvegicus vomeronasal 2 receptor, 38 (Vom2r38), mRNA [NM_001099476] | -3.53 | 1.08 |
| A_64_P053837 | Mc5r | ref\|Rattus norvegicus melanocortin 5 receptor (Mc5r), mRNA [NM_013182] | -3.54 | -1.67 |
| A_64_P075229 | Apba2 | ref\|Rattus norvegicus amyloid beta (A4) precursor protein-binding, family A, member 2 (Apba2), mRNA [NM_031780] | -3.54 | -1.43 |
| A_44_P384066 | G6pc | ref\|Rattus norvegicus glucose-6-phosphatase, catalytic subunit (G6pc), mRNA [NM_013098] | -3.54 | -1.83 |
| A_64_P106460 | Hoxc5 | ref\|Rattus norvegicus homeo box C5 (Hoxc5), mRNA [NM_001108116] | -3.54 | 1.10 |
| A_64_P092299 | Olr1666 | ref\|Rattus norvegicus olfactory receptor 1666 (Olr1666), mRNA [NM_001000108] | -3.59 | -1.91 |
| A_64_P109297 | Olr87 | ref\|Rattus norvegicus olfactory receptor 87 (Olr87), mRNA [NM_001000543] | -3.59 | 2.07 |
| A_64_P151146 | Iqub | ref\|Rattus norvegicus IQ motif and ubiquitin domain containing (Iqub), mRNA [NM_001034130] | -3.61 | -1.42 |
| A_42_P515346 | Map1a | ref\|Rattus norvegicus microtubule-associated protein 1A (Map1a), mRNA [NM_030995] | -3.63 | -1.69 |
| A_44_P255305 | Rgs17 | ref\|Rattus norvegicus regulator of G-protein signaling 17 (Rgs17), mRNA [NM_001107459] | -3.63 | 1.22 |
| A_44_P288548 | Prex1 | ref\|Rattus norvegicus phosphatidylinositol-3,4,5-trisphosphate-dependent Rac exchange factor 1 (Prex1), mRNA [NM_001135718] | -3.63 | -1.41 |
| A_44_P508566 | Olfml2b | ref\|Rattus norvegicus olfactomedin-like 2B (Olfml2b), mRNA [NM_001107195] | -3.63 | -1.27 |
| A_44_P114788 | ENSRNOT00000007358 | ens\|A disintegrin-like and metallopeptidase (Reprolysin type) with thrombospondin type 1 motif, 8 (Predicted)Uncharacterized protein [Source:UniProtKB/TrEMBL;Acc:D3ZNJ3] [ENSRNOT00000007358] | -3.63 | -1.45 |
| A_44_P105377 | Cenpb | ens\|Uncharacterized protein [Source:UniProtKB/TrEMBL;Acc:D4A5E3] [ENSRNOT00000002929] | -3.66 | 1.21 |
| A_64_P148806 | Ppef2 | ref\|Rattus norvegicus protein phosphatase, EF hand calcium-binding domain 2 (Ppef2), mRNA [NM_001107210] | -3.68 | 1.50 |
| A_64_P085971 | Arntl2 | ref\|Rattus norvegicus aryl hydrocarbon receptor nuclear translocator-like 2 (Arntl2), mRNA [NM_133391] | -3.70 | -2.88 |
| A_44_P1052046 | Abi3bp | ens\|Uncharacterized protein [Source:UniProtKB/TrEMBL;Acc:F1M0R2] [ENSRNOT00000049859] | -3.71 | 1.00 |
| A_64_P106760 | Tas2r144 | ref\|Rattus norvegicus taste receptor, type 2, member 144 (Tas2r144), mRNA [NM_001025150] | -3.75 | 1.06 |
| A_44_P500975 | Olr1639 | ref\|Rattus norvegicus olfactory receptor 1639 (Olr1639), mRNA [NM_001000833] | -3.79 | 1.05 |
| A_64_P160550 | Gpr171 | ref\|Rattus norvegicus G protein-coupled receptor 171 (Gpr171), mRNA [NM_001109510] | -3.79 | 1.51 |
| A_64_P123860 | Sh3bp2 | ref\|Rattus norvegicus SH3-domain binding protein 2 (Sh3bp2), mRNA [NM_001100684] | -3.80 | -1.01 |
| A_64_P078303 | Angpt4 | ref\|Rattus norvegicus angiopoietin 4 (Angpt4), mRNA [NM_001106526] | -3.82 | -1.23 |
| A_64_P033337 | Olr1198 | ref\|Rattus norvegicus olfactory receptor 1198 (Olr1198), mRNA [NM_001000436] | -3.83 | -1.30 |
| A_44_P298465 | Gcg | ref\|Rattus norvegicus glucagon (Gcg), mRNA [NM_012707] | -3.85 | -2.38 |
| A_44_P266833 | Syt3 | ref\|Rattus norvegicus synaptotagmin III (Syt3), mRNA [NM_019122] | -3.85 | 1.50 |
| A_44_P222124 | Tssk3 | ref\|Rattus norvegicus testis-specific serine kinase 3 (Tssk3), mRNA [NM_001007650] | -3.89 | -1.18 |
| A_44_P512874 | Cth | ref\|Rattus norvegicus cystathionase (cystathionine gamma-lyase) (Cth), mRNA [NM_017074] | -3.89 | -2.08 |
| A_44_P387308 | Adamtsl4 | ref\|Rattus norvegicus ADAMTS-like 4 (Adamtsl4), mRNA [NM_001034012] | -3.91 | 1.25 |
| A_64_P015637 | Cdhr4 | ref\|PREDICTED: Rattus norvegicus cadherin-related family member 4, transcript variant 2 (Cdhr4), mRNA [XM_002727137] | -3.92 | 1.09 |
| A_44_P277058 | Ccdc150 | ref\|Rattus norvegicus coiled-coil domain containing 150 (Ccdc150), mRNA [NM_001191806] | -3.94 | -2.44 |
| A_64_P067866 | Tmem92 | ref\|PREDICTED: Rattus norvegicus transmembrane protein 92 (Tmem92), mRNA [XM_001075148] | -3.94 | -1.95 |
| A_64_P144944 | Dab2 | ref\|Rattus norvegicus disabled 2, mitogen-responsive phosphoprotein (Dab2), mRNA [NM_024159] | -3.95 | -1.67 |
| A_44_P531611 | Mcf2l | ref\|Rattus norvegicus MCF.2 cell line derived transforming sequence-like (Mcf2l), mRNA [NM_053951] | -4.00 | 1.02 |
| A_64_P093847 | Olr1590 | ref\|Rattus norvegicus olfactory receptor 1590 (Olr1590), mRNA [NM_001000995] | -4.04 | -1.49 |
| A_64_P037388 | Rnf6 | ref\|Rattus norvegicus ring finger protein (C3H2C3 type) 6 (Rnf6), mRNA [NM_001107118] | -4.05 | 1.07 |
| A_64_P008371 | Olr811 | ref\|Rattus norvegicus olfactory receptor 811 (Olr811), mRNA [NM_001000848] | -4.06 | 1.26 |
| A_44_P388801 | Olr1583 | ref\|Rattus norvegicus olfactory receptor 1583 (Olr1583), mRNA [NM_001000080] | -4.07 | 1.44 |
| A_64_P040605 | Svs4 | ref\|Rattus norvegicus seminal vesicle secretory protein 4 (Svs4), mRNA [NM_012662] | -4.10 | -3.25 |
| A_44_P433851 | Hs3st5 | ref\|Rattus norvegicus heparan sulfate (glucosamine) 3-O-sulfotransferase 5 (Hs3st5), mRNA [NM_001106392] | -4.12 | 1.00 |
| A_43_P18716 | Ccdc78 | ens\|Putative uncharacterized protein [Source:UniProtKB/TrEMBL;Acc:B4F794] [ENSRNOT00000020363] | -4.12 | -2.41 |
| A_44_P121167 | Marcks | ref\|Rattus norvegicus myristoylated alanine rich protein kinase C substrate (Marcks), mRNA [NM_001271090] | -4.15 | 1.25 |
| A_64_P075357 | Cyr61 | ref\|Rattus norvegicus cysteine-rich, angiogenic inducer, 61 (Cyr61), mRNA [NM_031327] | -4.16 | -1.49 |
| A_64_P066045 | Kif26b | ref\|Rattus norvegicus kinesin family member 26B (Kif26b), mRNA [NM_001109079] | -4.19 | -1.19 |
| A_64_P375942 | Ptgs1 | ref\|Rattus norvegicus prostaglandin-endoperoxide synthase 1 (Ptgs1), mRNA [NM_017043] | -4.20 | -1.36 |
| A_44_P442645 | Olr1449 | ref\|Rattus norvegicus olfactory receptor 1449 (Olr1449), mRNA [NM_001000775] | -4.20 | -1.25 |
| A_64_P157964 | Vom1r49 | ref\|Rattus norvegicus vomeronasal 1 receptor 49 (Vom1r49), mRNA [NM_001008920] | -4.24 | 1.41 |
| A_64_P104268 | RT1-M1-5 | ref\|Rattus norvegicus RT1 class I, M1, gene 5 (RT1-M1-5), mRNA [NM_001168332] | -4.24 | 1.43 |
| A_64_P042107 | Pax6 | ref\|Rattus norvegicus paired box 6 (Pax6), mRNA [NM_013001] | -4.26 | -1.44 |
| A_64_P034793 | Olr1558 | ref\|Rattus norvegicus olfactory receptor 1558 (Olr1558), mRNA [NM_001000725] | -4.26 | -1.80 |
| A_43_P23055 | Spata3 | ref\|Rattus norvegicus spermatogenesis associated 3 (Spata3), mRNA [NM_001108805] | -4.27 | -2.00 |
| A_64_P015451 | Nanog | ref\|Rattus norvegicus Nanog homeobox (Nanog), mRNA [NM_001100781] | -4.27 | 1.32 |
| A_64_P085013 | Ksr2 | ens\|Uncharacterized protein [Source:UniProtKB/TrEMBL;Acc:F1LY04] [ENSRNOT00000046190] | -4.28 | -1.57 |
| A_44_P452186 | Epm2a | ens\|Laforin [Source:UniProtKB/Swiss-Prot;Acc:Q91XQ2] [ENSRNOT00000061930] | -4.29 | -2.32 |
| A_43_P23115 | Inmt | ref\|Rattus norvegicus indolethylamine N-methyltransferase (Inmt), mRNA [NM_001109022] | -4.29 | -1.35 |
| A_64_P052007 | Lef1 | ref\|Rattus norvegicus lymphoid enhancer binding factor 1 (Lef1), mRNA [NM_130429] | -4.30 | -1.75 |
| A_44_P131311 | Abca9 | ens\|Uncharacterized protein [Source:UniProtKB/TrEMBL;Acc:D3ZU16] [ENSRNOT00000005775] | -4.34 | -1.82 |
| A_43_P22854 | Dzip3 | ens\|Similar to Ubiquitin ligase protein DZIP3 (DAZ-interacting protein 3 homolog) (Predicted)Uncharacterized protein [Source:UniProtKB/TrEMBL;Acc:D3ZY61] [ENSRNOT00000002678] | -4.34 | -1.56 |
| A_44_P117142 | Vwa5b1 | ref\|Rattus norvegicus von Willebrand factor A domain containing 5B1 (Vwa5b1), mRNA [NM_001107988] | -4.34 | 1.51 |
| A_64_P011301 | Ugt2b17 | ref\|Rattus norvegicus UDP glucuronosyltransferase 2 family, polypeptide B17 (Ugt2b17), mRNA [NM_153314] | -4.36 | -2.01 |
| A_64_P006207 | Oxgr1 | ref\|Rattus norvegicus oxoglutarate (alpha-ketoglutarate) receptor 1 (Oxgr1), mRNA [NM_207588] | -4.42 | -2.05 |
| A_44_P807861 | Six4 | ref\|Rattus norvegicus SIX homeobox 4 (Six4), mRNA [NM_001106739] | -4.50 | 1.71 |
| A_64_P129720 | Cacng3 | ref\|Rattus norvegicus calcium channel, voltage-dependent, gamma subunit 3 (Cacng3), mRNA [NM_080691] | -4.51 | -1.04 |
| A_64_P007822 | Fezf1 | ref\|Rattus norvegicus Fez family zinc finger 1 (Fezf1), mRNA [NM_001109224] | -4.59 | -6.91 |
| A_44_P184578 | Olr1417 | ref\|Rattus norvegicus olfactory receptor 1417 (Olr1417), mRNA [NM_001001002] | -4.66 | -1.39 |
| A_64_P082067 | Ifit3 | ref\|Rattus norvegicus interferon-induced protein with tetratricopeptide repeats 3 (Ifit3), mRNA [NM_001007694] | -4.66 | 4.95 |
| A_64_P015168 | Ptprt | ref\|Rattus norvegicus protein tyrosine phosphatase, receptor type, T (Ptprt), mRNA [NM_001108603] | -4.67 | 1.00 |
| A_44_P539359 | Mmp28 | ref\|Rattus norvegicus matrix metallopeptidase 28 (Mmp28), mRNA [NM_001079888] | -4.70 | -1.44 |
| A_64_P135238 | Frmd6 | ref\|Rattus norvegicus FERM domain containing 6 (Frmd6), mRNA [NM_001271054] | -4.77 | -1.32 |
| A_44_P821875 | Ly49i7 | ref\|Rattus norvegicus immunoreceptor Ly49i7 (Ly49i7), mRNA [NM_001009500] | -4.77 | -1.43 |
| A_64_P131477 | Twist2 | ref\|Rattus norvegicus twist basic helix-loop-helix transcription factor 2 (Twist2), mRNA [NM_021691] | -4.80 | 1.00 |
| A_44_P360650 | Olr1058 | ref\|Rattus norvegicus olfactory receptor 1058 (Olr1058), mRNA [NM_001000066] | -4.85 | 1.05 |
| A_44_P116660 | Cd209b | ref\|Rattus norvegicus CD209b antigen (Cd209b), mRNA [NM_001170397] | -4.90 | -1.20 |
| A_44_P450075 | Ifit1lb | ens\|Uncharacterized protein [Source:UniProtKB/TrEMBL;Acc:F1M375] [ENSRNOT00000054756] | -4.95 | -1.16 |
| A_64_P101504 | Defb26 | ref\|Rattus norvegicus defensin beta 26 (Defb26), mRNA [NM_001037506] | -5.05 | -1.12 |
| A_64_P099938 | Olr652 | ref\|Rattus norvegicus olfactory receptor 652 (Olr652), mRNA [NM_001000638] | -5.09 | -1.48 |
| A_44_P548857 | Cadm2 | ref\|Rattus norvegicus cell adhesion molecule 2 (Cadm2), mRNA [NM_001047102] | -5.10 | 1.27 |
| A_64_P138984 | Rhox10 | ref\|Rattus norvegicus reproductive homeobox 10 (Rhox10), mRNA [NM_001037581] | -5.11 | 1.00 |
| A_44_P437896 | Bdnf | ref\|Rattus norvegicus brain-derived neurotrophic factor (Bdnf), transcript variant 2, mRNA [NM_012513] | -5.14 | 1.00 |
| A_44_P403396 | Snx33 | ref\|Rattus norvegicus sorting nexin 33 (Snx33), mRNA [NM_001127488] | -5.18 | 1.43 |
| A_64_P100629 | Olr783 | ref\|Rattus norvegicus olfactory receptor 783 (Olr783), mRNA [NM_001000376] | -5.25 | -3.07 |
| A_64_P030075 | Cnga1 | ref\|Rattus norvegicus cyclic nucleotide gated channel alpha 1 (Cnga1), mRNA [NM_053497] | -5.30 | 1.10 |
| A_44_P762856 | Mgarp | ref\|Rattus norvegicus mitochondria-localized glutamic acid-rich protein (Mgarp), nuclear gene encoding mitochondrial protein, mRNA [NM_001109556] | -5.33 | -1.76 |
| A_64_P237860 | Dmbt1 | ref\|PREDICTED: Rattus norvegicus deleted in malignant brain tumors 1 (Dmbt1), mRNA [XM_003748997] | -5.37 | -2.40 |
| A_44_P489330 | Olr1195 | ref\|Rattus norvegicus olfactory receptor 1195 (Olr1195), mRNA [NM_001001081] | -5.38 | -2.15 |
| A_64_P098532 | Lair1 | ref\|Rattus norvegicus leukocyte-associated immunoglobulin-like receptor 1 (Lair1), mRNA [NM_001029928] | -5.40 | -1.51 |
| A_64_P139962 | Olr527 | ref\|Rattus norvegicus olfactory receptor 527 (Olr527), mRNA [NM_001000675] | -5.60 | 1.17 |
| A_64_P098399 | Olr725 | ref\|Rattus norvegicus olfactory receptor 725 (Olr725), mRNA [NM_001000514] | -5.72 | 1.00 |
| A_44_P241909 | Epha7 | ref\|Rattus norvegicus Eph receptor A7 (Epha7), mRNA [NM_134331] | -5.76 | 1.21 |
| A_44_P138790 | Cirbp | ref\|Rattus norvegicus cold inducible RNA binding protein (Cirbp), mRNA [NM_031147] | -5.77 | 1.51 |
| A_64_P135479 | Olr1079 | ref\|Rattus norvegicus olfactory receptor 1079 (Olr1079), mRNA [NM_001000711] | -5.91 | -1.54 |
| A_44_P111816 | Adcy4 | ref\|Rattus norvegicus adenylate cyclase 4 (Adcy4), mRNA [NM_019285] | -5.91 | 1.43 |
| A_44_P172632 | Gpr1 | ref\|Rattus norvegicus G protein-coupled receptor 1 (Gpr1), mRNA [NM_012961] | -5.95 | -1.79 |
| A_44_P130968 | Tmx4 | ref\|Rattus norvegicus thioredoxin-related transmembrane protein 4 (Tmx4), mRNA [NM_001100529] | -6.11 | -1.01 |
| A_64_P136894 | Omd | ref\|Rattus norvegicus osteomodulin (Omd), mRNA [NM_031817] | -6.15 | 1.00 |
| A_64_P021043 | Pcsk9 | ref\|Rattus norvegicus proprotein convertase subtilisin/kexin type 9 (Pcsk9), mRNA [NM_199253] | -6.19 | -18.87 |
| A_64_P031945 | Hmgb1 | ref\|Rattus norvegicus high mobility group box 1 (Hmgb1), mRNA [NM_012963] | -6.20 | -1.02 |
| A_64_P059208 | Tcrb | gb\|Rat T-cell receptor variable region-beta 8.5 mRNA, 5' end. [M58627] | -6.26 | -3.26 |
| A_44_P400346 | Stab2 | gb\|Rattus norvegicus hyaluronan receptor for endocytosis HARE precursor, mRNA, partial cds. [AY007370] | -6.34 | -1.89 |
| A_64_P017841 | Olr722 | ref\|Rattus norvegicus olfactory receptor 722 (Olr722), mRNA [NM_001000572] | -6.43 | 1.00 |
| A_64_P016190 | Rictor | ens\|Uncharacterized protein [Source:UniProtKB/TrEMBL;Acc:F1M4J0] [ENSRNOT00000029565] | -6.80 | -2.25 |
| A_44_P133024 | Pabpc6 | ref\|Rattus norvegicus poly(A) binding protein, cytoplasmic 6 (Pabpc6), mRNA [NM_001106208] | -7.05 | 1.00 |
| A_43_P12995 | Kcns1 | ref\|Rattus norvegicus potassium voltage-gated channel, delayed-rectifier, subfamily S, member 1 (Kcns1), mRNA [NM_053954] | -7.12 | -1.42 |
| A_64_P066231 | Olr778 | ref\|Rattus norvegicus olfactory receptor 778 (Olr778), mRNA [NM_001000607] | -7.14 | 1.58 |
| A_64_P130808 | Olr136 | ref\|Rattus norvegicus olfactory receptor 136 (Olr136), mRNA [NM_001000160] | -7.14 | 1.59 |
| A_64_P143121 | Vom2r51 | ref\|Rattus norvegicus vomeronasal 2 receptor, 51 (Vom2r51), mRNA [NM_001099510] | -7.75 | -1.20 |
| A_43_P15460 | Cntn4 | ref\|Rattus norvegicus contactin 4 (Cntn4), mRNA [NM_053879] | -7.94 | 1.00 |
| A_44_P760567 | Pramel1 | ref\|Rattus norvegicus preferentially expressed antigen in melanoma-like 1 (Pramel1), mRNA [NM_001109617] | -7.96 | 1.02 |
| A_64_P034794 | Olr1458 | ref\|Rattus norvegicus olfactory receptor 1458 (Olr1458), mRNA [NM_001000021] | -7.98 | 1.00 |
| A_64_P056791 | Phf14 | ref\|Rattus norvegicus PHD finger protein 14 (Phf14), mRNA [NM_001110492] | -8.37 | 1.09 |
| A_44_P121586 | Tpte2 | ref\|Rattus norvegicus transmembrane phosphoinositide 3-phosphatase and tensin homolog 2 (Tpte2), mRNA [NM_001108877] | -8.89 | 1.00 |
| A_42_P764365 | Mpp4 | ref\|Rattus norvegicus membrane protein, palmitoylated 4 (MAGUK p55 subfamily member 4) (Mpp4), mRNA [NM_021265] | -10.96 | -1.22 |
| A_64_P016439 | Olr756 | ref\|Rattus norvegicus olfactory receptor 756 (Olr756), mRNA [NM_001000614] | -12.33 | -1.91 |
| A_64_P132559 | Btg4 | ref\|Rattus norvegicus B-cell translocation gene 4 (Btg4), mRNA [NM_001013176] | -12.68 | -1.20 |
| A_64_P045315 | Kbtbd4 | ref\|Rattus norvegicus kelch repeat and BTB (POZ) domain containing 4 (Kbtbd4), mRNA [NM_001107746] | -13.70 | 1.40 |
| A_64_P039742 | Acot6 | ens\|RCG20716Uncharacterized protein [Source:UniProtKB/TrEMBL;Acc:D3ZSE3] [ENSRNOT00000058101] | -13.80 | -1.72 |
| A_43_P15558 | Htr1b | ref\|Rattus norvegicus 5-hydroxytryptamine (serotonin) receptor 1B, G protein-coupled (Htr1b), mRNA [NM_022225] | -15.07 | 1.00 |
| A_44_P400338 | F11 | ref\|Rattus norvegicus coagulation factor XI (F11), mRNA [NM_001047848] | -16.37 | 1.11 |
| A_64_P054466 | Bhlhb9 | ref\|Rattus norvegicus basic helix-loop-helix domain containing, class B, 9 (Bhlhb9), mRNA [NM_207611] | -18.23 | -3.06 |
| A_64_P080791 | Olr413 | ref\|Rattus norvegicus olfactory receptor 413 (Olr413), mRNA [NM_001000826] | -19.20 | 1.10 |
| A_43_P12433 | Grid2 | ref\|Rattus norvegicus glutamate receptor, ionotropic, delta 2 (Grid2), mRNA [NM_024379] | -19.23 | -1.82 |
